# Elucidating the transactivation domain of the pleiotropic transcription factor Myrf

**DOI:** 10.1038/s41598-018-31477-4

**Published:** 2018-08-30

**Authors:** Jin-ok Choi, Chuandong Fan, Dongkyeong Kim, Mohamed Sharif, Hongjoo An, Yungki Park

**Affiliations:** Hunter James Kelly Research Institute, Department of Biochemistry, Jacobs School of Medicine and Biomedical Sciences, SUNY Buffalo, Buffalo, NY 14203 USA

## Abstract

Myrf is a newly discovered membrane-bound transcription factor that plays an essential role in as diverse organisms as human, worm, and slime mold. Myrf is generated as a type-II membrane protein in the endoplasmic reticulum (ER). It forms homo-oligomers to undergo auto-cleavage that releases Myrf N-terminal fragment from the ER membrane as a homo-trimer. The homo-trimer of Myrf N-terminal fragments enters the nucleus and binds the Myrf motif to activate transcription. Despite its prominent role as a transcriptional activator, little is known about the transactivation domain of Myrf. Here, we report that the N-terminal-most (NTM) domain of Myrf is required for transcriptional activity and, when fused to a Gal4 fragment, enables it to activate transcription. The transactivation function of the NTM domain did not require homo-trimerization. We also discovered that the NTM domain can be sumoylated at three lysine residues (K123, K208, and K276), with K276 serving as the main acceptor. K276 sumoylation repressed the transactivation function of the NTM domain without affecting the stability or nuclear localization of Myrf N-terminal fragment. In sum, this study identifies the NTM domain as the transactivation domain of Myrf and the potential regulatory impact of its K276 sumoylation.

## Introduction

Myrf (Myelin regulatory factor) is a newly discovered pleiotropic membrane-bound transcription factor^1–4^. In the mammalian central nervous system (CNS), the expression of Myrf is restricted to oligodendrocytes (OLs) that make myelin sheaths^1^. OL-specific deletion of Myrf blocks the maturation of OLs, resulting in severe dysmyelination throughout the CNS^1^. Myrf is also essential for the life-long maintenance and regeneration of CNS myelin^5,6^ and its plasticity that underlies learning^7^. These studies left the impression that Myrf is a “myelin” transcription factor. However, Myrf orthologs are also found in organisms without myelin, where they were required for molt and synaptic plasticity (*Caenorhabditis elegans*)^8,9^ and prestalk cell differentiation (*Dictyostelium*)^4,10^. Consistent with the important roles of Myrf going beyond CNS myelination, germline deletion of Myrf in mice caused embryonic lethality^1^, and Myrf coding variants have been implicated in the pathogenesis of congenital heart disease and congenital diaphragmatic hernia as well as encephalopathy^11–14^.

**Figure 1:**
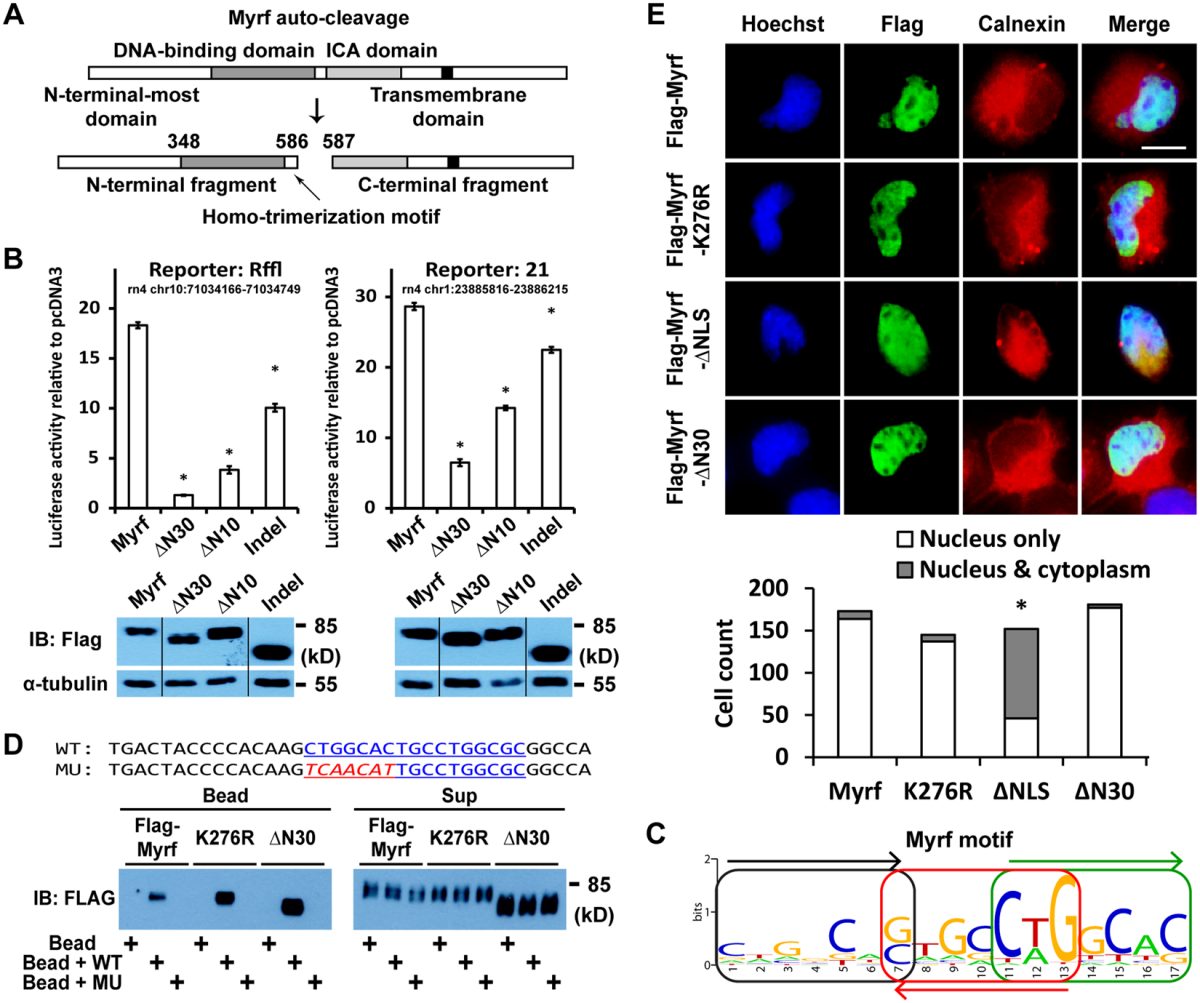
The N-terminal-most (NTM) domain of Myrf is required for transcriptional activity. (**A**) The auto-cleavage scheme of Myrf. (**B**) The transcriptional activity of wild-type Myrf N-terminal fragment was compared with that of mutant ones by luciferase assay. The luciferase activity of pcDNA3 was set to 1, and the reported values are means and standard errors. *p < 0.001 by two-tailed unpaired Student’s *t* test with Bonferroni correction. The luciferase assay samples were subsequently analysed by Western blot to examine protein expression levels. The grouping of blots cropped from different parts of the same gel is indicated by dividing lines. The full blots are shown in Supplementary Figure 1A. (**C**) The 17-base pair long Myrf motif that is recognized by the homo-trimer of Myrf N-terminal fragments. (**D**) DNA pulldown assay showed that Myrf-ΔN30 N-terminal fragment correctly distinguishes the Myrf motif (WT) from a decoy version (MU). The full blot is shown in Supplementary Figure 1B. (**E**) The subcellular localization of Myrf N-terminal fragment was determined by immunofluorescence in Oli-neu cells. A representative example is shown for each Myrf construct, and quantitative analysis is shown below the images. Scale bar, 10 µm. Myrf-ΔNLS N-terminal fragment is frequently found outside of the nucleus compared with the wild-type one (*p ≈ 0 by cumulative binomial distribution with Bonferroni correction). IB: immunoblotting.

We and others have reported that Myrf is generated as a type-II membrane protein in the endoplasmic reticulum (ER)^2,3^. The Intramolecular Chaperone Auto-processing (ICA) domain of Myrf induces its homo-oligomerization in the ER membrane, and homo-oligomeric Myrf undergoes an auto-cleavage reaction to release its N-terminal fragment from the ER membrane as a homo-trimer (Fig. 1A)^15^. Remarkably, this auto-cleavage mechanism is conserved from human to slime mold^4,9^. In mammals, the homo-trimeric complex of Myrf N-terminal fragments appears to enter the nucleus immediately upon auto-cleavage to work as a transcription factor^2,3^. In contrast, the nuclear entry step seems to be under regulation for the worm and slime mold orthologs of Myrf^4,8,9^. A recent structural study confirmed the homo-trimerization status for Myrf N-terminal fragments^16^, and we found that homo-trimerization imparts DNA-binding specificity to Myrf N-terminal fragment^15^, which provides a rational explanation for the lethal phenotype of the *let-25* allele of *C*. *elegans*^8^.

Despite its prominent role as a transcriptional activator, little is known about the transactivation domain of Myrf. This knowledge would be essential to advance our understanding of the functional mechanisms of Myrf. This paper reports our elucidation of the transactivation domain of Myrf. We found that the N-terminal-most (NTM) domain of Myrf is required for transcriptional activity and can confer transcriptional activity to a Gal4 fragment. Further, our work shows that homo-trimerization is not critical to the transactivation function of the NTM domain. Thus, the NTM domain appears to function as an autonomous transactivator. Unexpectedly, we discovered that the NTM domain contains three consensus sumoylation motifs at K123, K208 and K276, and our biochemical analysis revealed that Myrf can be sumoylated at these lysine residues. We also found that K276 sumoylation represses the transactivation function of NTM. Overall, we elucidate the transactivation domain of Myrf and the potential regulatory impact of its K276 sumoylation.

## Results

### The N-terminal-most domain of Myrf is required for transcriptional activity

Upon auto-cleavage, the homo-trimer of Myrf N-terminal fragments is released from the ER membrane and enters the nucleus to function as a transcription factor. Myrf N-terminal fragment can be divided into three parts – the N-terminal-most (NTM) domain, the DNA-binding domain, and the homo-trimerization motif (Fig. 1A). Given this domain arrangement and the fact that Myrf N-terminal fragment works as a transcriptional activator^2,3,15^, the NTM domain is likely to work as a transactivation domain. NTM is proline-rich (76/348 residues are prolines). Bioinformatics analysis suggests that the NTM domain has few α helices and β strands and is largely disordered (data not shown). These sequence and structural features are often found in transactivation domains^17,18^, supporting our hypothesis. To determine whether NTM is required for the transcriptional activity of Myrf N-terminal fragment, we gradually truncated its N terminus and assessed its impact on transcriptional activity. The regions of rat chr10:71034166-71034749 and chr1:23885816-23886215 (both in rn4) were identified as to be bound by Myrf in differentiating OLs^2^, and we cloned them into pGL3-promoter to generate the Myrf luciferase reporters Rffl and 21, respectively. The expression of Myrf effectively increased the reporter activity of Rffl and 21 in Oli-neu cells^19^ (a widely used mouse OL cell line) (Fig. 1B). Strikingly, the deletion of the N-terminal 30 residues was sufficient to abolish transcriptional activity (ΔN30, Fig. 1B). Similarly, the loss of the N-terminal 10 residues was highly disruptive (ΔN10, Fig. 1B). We generated another mutant in which the region spanning residues 117-243 is deleted (Indel). The transcriptional activity of Indel was significantly lower than that of wild-type Myrf (Fig. 1B). Western blot analysis of the luciferase assay samples showed that Myrf N-terminal fragments were expressed at a similar level across the Myrf constructs (Fig. 1B). These results show that NTM is required for the transcriptional activity of Myrf N-terminal fragment.

### NTM disruption affects neither the DNA binding nor nuclear localization of Myrf N-terminal fragment

To elucidate why the NTM domain is required for the transcriptional activity of Myrf N-terminal fragment, we determined whether Myrf-ΔN30 N-terminal fragment correctly binds to the Myrf motif, a 17-base pair long DNA motif that is recognized by the homo-trimer of Myrf N-terminal fragments for transcriptional activation (Fig. 1C)^15^. Flag-Myrf and Flag-Myrf-ΔN30 were expressed in HEK293FT cells, and cell lysates were incubated with magnetic beads coated with the Myrf motif incidence found in *Rffl* (WT in Fig. 1D) or its mutant version (MU in Fig. 1D). The incubation mixture was separated into the bead and sup fractions. As previously reported^2,15^, immunoblotting of the bead fractions showed that the homo-trimer of Myrf N-terminal fragments did not bind to blank beads and correctly distinguished the Myrf motif from a decoy version (Fig. 1D). Myrf-ΔN30 N-terminal fragments bound to the Myrf motif with the same strength and specificity as the wild-type counterpart (Fig. 1D). Immunoblotting of the sup fractions showed that Myrf N-terminal fragments were present at a similar level across each set of the binding reactions.

To determine whether Myrf-ΔN30 N-terminal fragment is correctly localized to the nucleus, we performed immunofluorescence experiments. Flag-Myrf and Flag-Myrf-ΔN30 were transfected into Oli-neu cells, and the nuclear localization of their N-terminal fragments was determined by immunofluorescence. In this experiment, Myrf-ΔNLS, a mutant where a previously characterized nuclear localization signal (K_254_KRK_257_)^2,3^ is replaced by four alanine residues, was used as a control. We found that Myrf-ΔN30 N-terminal fragment was localized to the nucleus at a comparable rate to the wild-type one (Fig. 1E). As expected, Myrf-ΔNLS N-terminal fragments were frequently localized outside of the nucleus. These results demonstrate that Myrf-ΔN30 N-terminal fragment correctly recognizes the Myrf motif in the nucleus. In turn, this suggests that the NTM domain is required for the transcriptional activity of Myrf N-terminal fragment because it works as a transactivation domain.

### NTM is sufficient for transcriptional activation

The above experiments show that the NTM domain is necessary for transcriptional activation. As a complementary analysis, we sought to determine whether NTM is sufficient for transcriptional activation. To this end, we took advantage of the Gal4 fusion system^20^. In this system, a putative transactivation domain is fused to the N-terminal fragment of Gal4, a yeast transcription factor. Gal4 N-terminal fragment provides nuclear entry and DNA binding functionality. The fusion construct is co-transfected with 5xGAL4-TATA, a luciferase reporter equipped with a minimal promoter and upstream Gal4 binding sites^20^. If the putative transactivation domain is a *bona fide* transactivation domain, it may be able to recruit transcriptional co-activators and elicit a transcription response in this heterologous context. Importantly, the NTM domain works in the homo-trimer context for Myrf while Gal4 fusion proteins are known to work as obligatory homo-dimers^21^. So an additional boon of the Gal4 system for NTM is that it would help us to determine whether homo-trimerization is essential for the transactivation function of the NTM domain.

**Figure 2:**
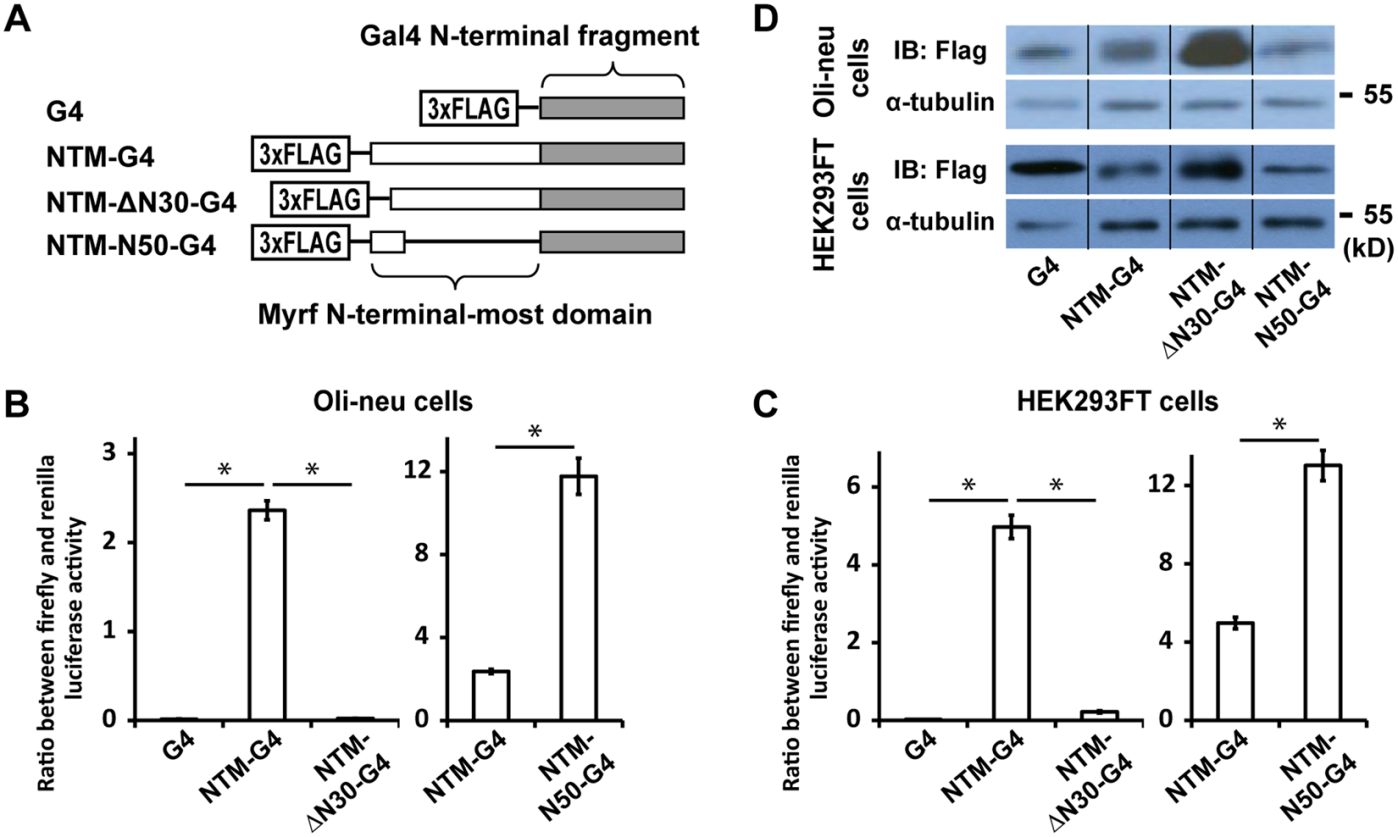
The NTM domain is sufficient for transcriptional activation. (**A**) NTM-G4 is a fusion between the NTM domain and Gal4 N-terminal fragment (G4). NTM-ΔN30-G4 is the same as NTM-G4 except that the N-terminal 30 residues of the NTM domain are deleted. NTM-N50-G4 is a fusion between the first 50 N-terminal residues of the NTM domain and G4. The transcriptional activity of G4, NTM-G4, NTM-ΔN30-G4, and NTM-N50-G4 was determined by luciferase assay in Oli-neu and HEK293FT cells in panels B and C, respectively. Shown are means and standard errors. *p < 0.01 by two-tailed unpaired Student’s *t* test with Bonferroni correction. (**D**) The luciferase assay samples of panels B and C were analysed by Western blot to examine protein expression levels. The grouping of blots cropped from different parts of the same gel is indicated by dividing lines. The full blots are shown in Supplementary Figure 2.

We prepared 4 constructs – G4, NTM-G4, NTM-ΔN30-G4, and NTM-N50-G4 (Fig. 2A). G4 is Gal4 N-terminal fragment alone and used as a control. NTM-G4 is G4 with NTM fused N-terminally. NTM-ΔN30-G4 is the same as NTM-G4 except that the N-terminal 30 residues of NTM are deleted. NTM-N50-G4 is a fusion between the N-terminal 50 residues of NTM and G4. All constructs were based on pcDNA3 with N-terminal Flag tags. These, together with 5xGAL4-TATA, were transfected into Oli-neu cells. We found that the NTM domain activates transcription in this heterologous context (NTM-G4 in Fig. 2B), demonstrating that NTM is sufficient for transcriptional activation, and that homo-trimerization is not absolutely required for it. The deletion of the N-terminal 30 residues completely abrogated transcriptional activation (NTM-ΔN30-G4 in Fig. 2B), as in the Myrf context. We also found that the N-terminal 50 residues of NTM are sufficient for transcriptional activation (NTM-N50-G4 in Fig. 2B). These results show that the N-terminal residues are essential for the autonomous transactivation function of the NTM domain. Western blot analysis of the luciferase assay samples ruled out the trivial possibility that the lack of transcriptional activity for NTM-ΔN30-G4 was due to its poor expression and that the enhanced transcriptional activity of NTM-N50-G4 was due to its higher expression (Fig. 2D). To test the generality of our conclusion, we repeated the luciferase assay with HEK293FT cells and obtained identical results (Fig. 2C,D). Overall, we conclude that NTM is an autonomous transactivation domain with its N-terminal residues playing a critical role.

### NTM can be sumoylated at K123, K208, and K276

**Figure 3:**
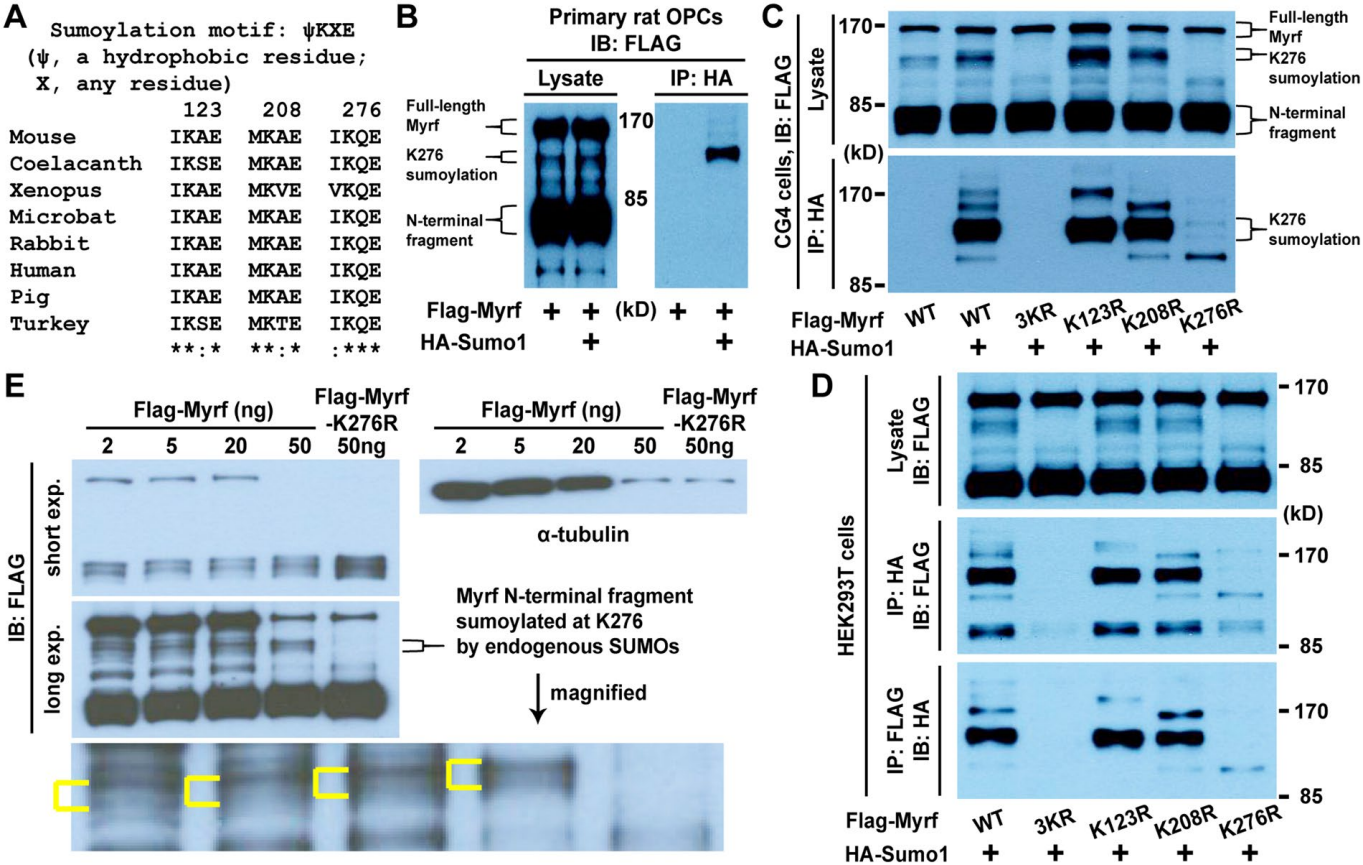
Myrf can be sumoylated at the three consensus sumoylation motifs in the NTM domain (K123, K208, and K276). (**A**) Three consensus sumoylation motifs in the NTM domain. (**B**) Immunoprecipitation with HA beads demonstrated that Myrf N-terminal fragment can be sumoylated in primary OLs. The full blot is shown in Supplementary Figure 3A. (**C**) Immunoprecipitation with HA beads revealed that K123, K208, and K276 serve as major SUMO acceptors for Myrf in CG4 cells. Further, it showed that Myrf K276 is sumoylated by endogenous SUMOs. The full blots are shown in Supplementary Figure 3B. (**D**) Immunoprecipitation with HA and FLAG beads revealed that K123, K208, and K276 serve as major SUMO acceptors for Myrf in HEK293FT cells. The full blots are shown in Supplementary Figure 3C. (**E**) Myrf is sumoylated at K276 by endogenous SUMOs even when it is not overexpressed. Flag-Myrf was transfected into Oli-neu cells in 35 mm dishes. Cell lysates were subject to immunoblot analysis. Myrf K276 sumoylation by endogenous SUMOs is delineated by yellow brackets in the magnified image. To equalize immunoblot signals, the amounts of cell lysates loaded were different, as shown by the α-tubulin immunoblot. The full blots are shown in Supplementary Figure 3D. IB: immunoblotting. IP: immunoprecipitation.

The activity, stability, and subcellular localization of transcription factors are often regulated by post-translational modifications. Sumoylation is one such mechanism, where small ubiquitin-related modifier (SUMO) proteins are covalently attached to the target protein^22–26^. The reversible sumoylation pathway starts with the sequential activation of SUMO proteins by SUMO E1 and E2 ligases in much the same way as ubiquitin is activated. An activated SUMO is then transferred to the target protein, usually with the aid of an E3 ligase. Conjugated SUMOs can be deconjugated by SUMO-specific proteases^24,25^. Survey of the amino acid sequence of the NTM domain revealed the presence of three consensus sumoylation motifs at K123, K208, and K276 (Fig. 3A), suggesting that NTM may undergo sumoylation. Further, the three lysine residues are well conserved across species, suggesting that sumoylation may modulate the transactivation function of the NTM domain. To determine whether Myrf can be sumoylated, we transfected Flag-Myrf and HA-Sumo1 (Sumo1 with an N-terminal HA tag) into primary OL precursor cells (OPCs) purified from rat pups by immunopanning^27^. Cell lysates were subject to immunoprecipitation with HA beads. When Flag-Myrf was expressed alone, it did not bind to HA beads (Fig. 3B). When coexpressed with HA-Sumo1, it did bind to HA beads, indicating that Myrf is conjugated with Sumo1 in primary OPCs cultured in the differentiation condition^27^. The same result was obtained with CG4 cells, a widely used rat OL cell line^28^ (Fig. 3C). Sumoylation is known to be unstable due to the ongoing activity of SUMO-specific proteases^22–24^. Presumably for this reason, we had to use a denaturing lysis buffer in these immunoprecipitation experiments (see Methods), strongly suggesting that Myrf and Sumo1 were covalently connected. To determine whether K123, K208, and K276 serve as SUMO acceptors for Myrf, we mutated them to arginine and repeated immunoprecipitation experiments. When all three lysine residues were mutated to arginine, sumoylation was completely abolished (3KR in Fig. 3C), indicating that the three lysine residues are the main SUMO acceptors. The mutation of individual lysine residues led to distinct changes in the sumoylation pattern (K123R, K208R, and K276R in Fig. 3C). Of the three lysine residues, K276 appeared to be sumoylated at the highest level. These results were fully reproduced in HEK293FT cells (Fig. 3D).

Western blot analysis of primary OL and CG4 cell lysates shows that overexpressed Myrf is sumoylated at K276 by endogenous SUMOs (Fig. 3B,C). We wondered whether this is an overexpression artifact. To address this issue, Oli-neu cells in 35 mm dish were transfected with a minute amount of Flag-Myrf plasmid (2 ng in Fig. 3E). Given that several hundred nanograms or more of plasmid are usually transfected for 35 mm dishes, this represents more than a 100-fold reduction. At this condition, overexpression was indeed effectively controlled, as indicated by the appearance of full-length uncleaved Myrf in the immunoblot (Fig. 3E). Homo-trimerization is a prerequisite for the auto-cleavage of Myrf. When Myrf is expressed at a high level, a majority of Myrf forms homo-trimers to undergo auto-cleavage, making it difficult to observe full-length uncleaved Myrf in immunoblots (even at 50 ng, Fig. 3E). As the expression level of Myrf goes down, homo-trimerization becomes more of a rate-limiting step, making it easy to observe full-length uncleaved Myrf in immunoblots (20 ng or less, Fig. 3E). Remarkably, Myrf K276 sumoylation by endogenous SUMOs, represented by a doublet (yellow brackets in the magnified image in Fig. 3E), was apparent at 2 ng. By overlapping the developed films, we confirmed that the doublet observed at the 2 ng condition exactly matches the one observed at the 50 ng condition, which was importantly absent for Flag-Myrf-K276R. Thus, Myrf is sumoylated at K276 by endogenous SUMOs even when it is not overexpressed, suggesting that endogenous Myrf undergoes sumoylation at K276. Unfortunately, we could not directly verify K276 sumoylation of endogenous Myrf because of the lack of suitable antibodies for Western blot analysis of Myrf N-terminal fragment.

### K276 sumoylation represses the transactivation function of NTM

**Figure 4:**
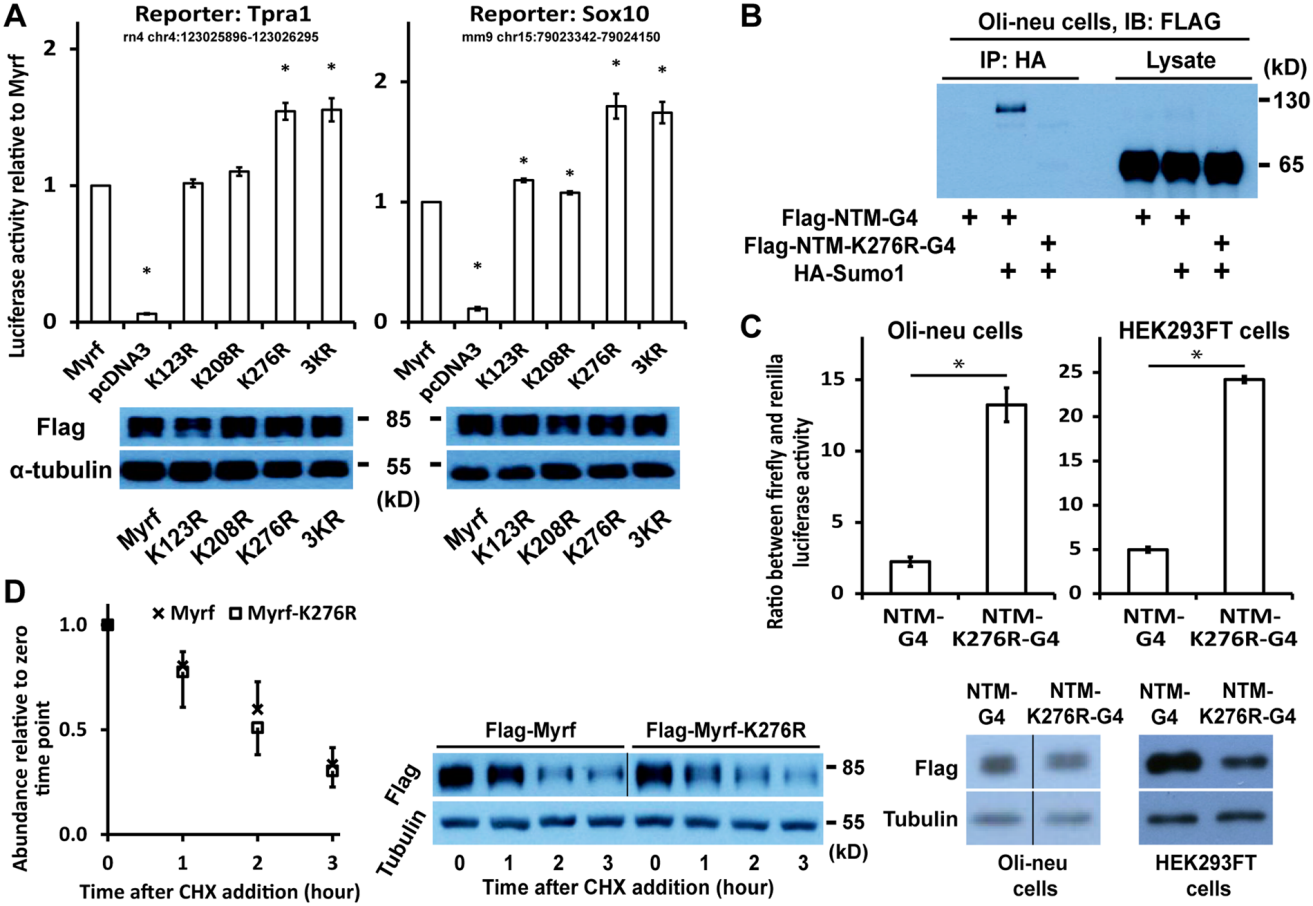
Myrf K276 sumoylation represses the transactivation function of NTM. (**A**) The transcriptional activity of wild-type Myrf was compared with that of mutant ones by luciferase assay in Oli-neu cells. The luciferase activity of Myrf was set to 1, and the reported values are means and standard errors. *p < 0.05 by two-tailed unpaired Student’s *t* test with Bonferroni correction. The luciferase assay samples were subsequently analysed by Western blot to check protein expression levels. The full blots are shown in Supplementary Figure 4A. (**B**) Immunoprecipitation experiments showed that K276 undergoes sumoylation in the Gal4 fusion context. The full blot is shown in Supplementary Figure 4B. (**C**) Luciferase assay showed that K276 sumoylation represses the transactivation function of NTM in the context of Gal4 fusion proteins. NTM-K276R-G4 is the same as NTM-G4 (Fig. 2A) except for the K276R mutation that prevents K276 sumoylation. Shown are means and standard errors. *p < 0.001 by two-tailed unpaired Student’s *t* test with Bonferroni correction. The luciferase assay samples were subsequently analysed by Western blot to check protein expression levels. The grouping of blots cropped from different parts of the same gel is indicated by dividing lines. The full blots are shown in Supplementary Figure 4C. (**D**) The K276R mutation does not affect the stability of Myrf N-terminal fragment. Oli-neu cells were transfected with Flag-Myrf or Flag-Myrf-K276R. Transfected cells were aliquoted into small dishes and subject to cycloheximide treatment (25 ug/ml) for different durations to determine the degradation rate of Myrf N-terminal fragment. The grouping of blots cropped from different parts of the same gel is indicated by the dividing line. As indicated in the full blot (Supplementary Figure 4D), the results for Myrf and Myrf-K276R are from different exposure times (2 min and less than 1 min, respectively). IB: immunoblotting. IP: immunoprecipitation.

We hypothesized that sumoylation may alter the transactivation function of the NTM domain. To test this hypothesis, we performed luciferase assay where the transcriptional activity of Myrf was compared with that of sumoylation mutants. The regions of rat chr4:123025896-123026295 and chr7:117175832-117176231 (both in rn4) were identified as to be bound by Myrf in differentiating OLs^2^, and we cloned the former and a mouse genomic fragment corresponding to the latter into pGL3-promoter to generate the Myrf luciferase reporters Tpra1 and Sox10, respectively. The expression of Myrf effectively increased the reporter activity of Tpra1 and Sox10 (Fig. 4A). For both reporters, the transcriptional activity of the K276R mutant was significantly higher than that of wild-type Myrf, which was also mirrored by the 3KR mutant (Fig. 4A). Western blot analysis of the luciferase assay samples showed similar protein expression levels. These results suggest that K276 sumoylation represses the transactivation function of the NTM domain. To corroborate this conclusion, we tested the effect of K276 sumoylation in the context of Gal4 fusion proteins. NTM-K276R-G4 is the same as NTM-G4 (Fig. 2A) except for the K276R mutation. Immunoprecipitation experiments confirmed that K276 is sumoylated in the NTM-G4 context as well (Fig. 4B). The replacement of K276 by arginine led to a 5-fold increase in the transactivation function of NTM (Fig. 4C). Western blot analysis of the luciferase assay samples revealed that NTM-G4 and NTM-K276R-G4 were expressed at a comparable level. Myrf K276 underwent sumoylation in all the cell types tested (HeLa, HEK293FT, Oli-neu and CG4 cells, and primary rat OPCs). This prompted the hypothesis that K276 sumoylation is a general (*i*.*e*., not oligodendrocyte-specific) regulatory mechanism that is hardwired into the NTM domain. Indeed, the luciferase assay results from Oli-neu cells were reproduced in HEK293FT cells (Fig. 4C). Importantly, we found that the K276R mutation alters neither the DNA-binding nor nuclear localization of Myrf N-terminal fragment (Fig. 1D,E). Consistent with the Western blot result in Fig. 4A, the K276R mutation did not affect the stability of Myrf N-terminal fragment (Fig. 4D). Taken together, we conclude that K276 sumoylation has the inherent property of repressing the autonomous transactivation function of the NTM domain.

## Discussion

Myrf is a pleiotropic membrane-bound transcription factor that plays an essential role in diverse organisms^1,5,7–10^. Upon auto-cleavage in the ER membrane, its N-terminal fragment enters the nucleus as a homo-trimer and binds to the Myrf motif to activate transcription^2–4,9,15^. Despite its prominent role as a transcriptional activator^2,15^, there is scant information on the transactivation domain of Myrf, and this knowledge would be necessary to better understand the functional mechanisms of Myrf. Our thorough analysis identifies the N-terminal-most (NTM) domain of Myrf as its transactivation domain. First, we found that the NTM domain is required for the transcriptional activity of Myrf N-terminal fragment, satisfying the necessity condition. Second, when fused to a Gal4 N-terminal fragment, NTM rendered it transcriptionally active, meeting the sufficiency requirement. Residues in the N terminus of the NTM domain seem especially important for its transactivation function because their deletion almost completely abolished it. Corroborating this observation, the fusion of NTM to the Gal4 N-terminal fragment worked only when its N-terminal residues were intact. Since the deletion of the N-terminal residues did not affect the stability, DNA binding, and nuclear localization of Myrf N-terminal fragment, we think that the N-terminal residues of the NTM domain are critical to the physical interaction between Myrf N-terminal fragment and an unknown co-activator. The unknown co-activator must be a general factor that is expressed in both Oli-neu and HEK293FT cells because the deletion of the N-terminal residues had the same effect in both cell lines. Further studies will be required to identify the unknown co-activator and its role in the transcriptional activity of Myrf N-terminal fragment. Our study also shows that homo-trimerization is not essential for the transactivation function of NTM, unlike the DNA-binding and ICA domains of Myrf whose function depends on homo-trimerization^3,15,29,30^. However, homo-trimerization may enhance the transactivation function of the NTM domain either quantitatively (*i*.*e*., simply by increasing its local concentration, facilitating the interaction with the unknown co-activator) or qualitatively (*i*.*e*., by enabling a new mode of interaction with the co-activator).

Unexpectedly, we found three consensus sumoylation motifs in the NTM domain (K123, K208, and K276), which prompted us to hypothesize that NTM may be sumoylated, and that sumoylation may modulate its transactivation function. Indeed, our biochemical assay demonstrated that Myrf N-terminal fragment can be sumoylated at the three lysine residues. Further, we found that the sumoylation of K276, the main sumoylation site, represses the transactivation function of NTM. The difference in the repressive effect among the three sumoylation sites may be due to the quantitative difference in the sumoylation level (K276 is the most sumoylated site and thus has the greatest effect) or the qualitative difference (K276 sumoylation is positioned such that it effectively interferes with the interaction between Myrf N-terminal fragment and the transcriptional co-activator). K276 sumoylation reproducibly led to an increase of 30-40 kD in the apparent molecular weight. Sumo2/3 are known to form polymers through their internal sumoylation sites while Sumo1 works as a chain terminator due to the absence of such sites^25^. Thus, given that each SUMO molecule is about 10 kD, the polymer attached to Myrf K276 may consist of two or three internal Sumo2/3 molecules and one terminal Sumo1 molecule. This polymer may be bulky enough to disrupt the physical interaction between Myrf N-terminal fragment and the co-activator.

In line with the pleiotropic role of Myrf, ten Myrf genetic alleles (seven missense mutations, one nonsense mutation, one frameshift mutation, and one splice donor site mutation) have been implicated in the genetic risk of congenital heart disease, congenital diaphragmatic hernia, and encephalopathy with reversible myelin vacuolization^11–14^. Heart disorder and diaphragm hernia tend to co-occur, along with other malformations such as urogenital tract defects, lung hypoplasia, and intellectual disability. So it has been suggested that Myrf-implicated congenital anomalies represent a novel type of complex birth defect^11,12,14^, which is consistent with the essential role of Myrf in embryonic development^1^. The seven missense mutations are mapped to the DNA-binding and ICA domains of Myrf. No mutation in the NTM domain has yet been reported in association with human disease. However, sequencing studies in the future are likely to uncover disease-linked missense mutations in the NTM domain. In fact, the ExAC database reveals many rare Myrf missense mutations that hit highly conserved residues in NTM^31^, which may significantly disrupt its transactivation function. Given the indispensable role of Myrf in diverse organisms and cellular processes, our study that identifies NTM as the transactivation domain of Myrf and the potential regulatory impact of its K276 sumoylation lays an important ground work.

## Methods

### Constructs

A mouse Myrf cDNA that encodes the 1139-amino-acid-long isoform was kindly provided by Dr. Ben Emery^1,15^. The mouse Myrf cDNA was cloned into pcDNA3. The HA-Sumo1 plasmid was a gift from Guy Salvesen (Addgene plasmid #48966)^32^. All mutagenesis was carried out by a PCR-based method, and sequence information was verified by Sanger sequencing.

### Tissue Harvest and Cell Culture

Animal husbandry and tissue harvest were carried out in accordance with the guidelines and protocols of the University at Buffalo (SUNY Buffalo) Institutional Animal Care and Use Committee (IACUC, approved protocol #NA-Park2). Oligodendrocyte precursor cells (OPCs) were purified from rat pups of P7~P9 by immunopanning^27^. Primary OPCs, CG4 and Oli-neu cells were kept in a proliferative condition by supplementing the Sato media^27^ with PDGF (10 μg/mL), NT3 (1 μg/mL), CNTF (10 μg/mL), and NeuroCult™ SM1 Neuronal Supplement (only for Oli-neu cells). Primary OPCs, CG4 and Oli-neu cells were maintained in a humidified 8% CO_2_ incubator at 37 °C. HeLa and HEK293FT cells were cultured in Dulbecco’s modified Eagle’s medium supplemented with 10% fetal bovine serum and maintained in a humidified 5% CO_2_ incubator at 37 °C. Transient transfection was performed by using Lipofectamine 2000 as per the manufacturer’s instructions.

### Immuoprecipitation and Immunoblotting

Cells were lysed with a lysis buffer (1% SDS, 50 mM Tris pH 6.8, 2.5% beta-mercaptoethanol, protease inhibitor cocktail (Roche), and 2 mM PMSF). Cell lysate was boiled for 5 min and then sonicated with a bioruptor for 10 min. Cell lysate was centrifuged at 14,000 *g* for 10 min at 4 °C. Cleared cell lysate was mixed with 10 ul anti-HA agarose bead slurry (Sigma #E6779) or anti-FLAG M2 affinity agarose gel (Sigma #A2220) for immunoprecipitation for 2 hr at 4 °C. The mixture was centrifuged at 7000 *g* for 1 min. Beads were washed with the lysis buffer 5 times for a total of 1 hour. Samples were mixed with Laemmli sample buffer and boiled at 95 °C for 5 min. Upon electrophoresis, proteins were transferred to PVDF and probed with primary and HRP-conjugated secondary antibodies. Reagents used for immunoblotting are as follows: HRP conjugated FLAG antibody (Sigma #A8592, 1:5000), α-tubulin antibody (Sigma #T6199, 1:10000), anti-HA tag antibody, HRP-conjugated (Cell Signaling #2999, 1:5000), and goat anti-mouse antibody, HRP conjugate (ThermoFisher #A16072, 1:10000).

### Immunofluorescence

Cells were fixed with 4% formaldehyde and permeabilized with 0.1% Triton X-100. Upon blocking with 1% BSA, they were incubated with primary antibodies diluted in blocking buffer at 4 °C overnight, followed by incubation with fluorochrome-conjugated secondary antibodies. Fluorescence was visualized with a Leica DMi8 microscope with an ORCA-Flash4.0 sCMOS camera. Reagents used for immunofluorescence are as follows: FLAG antibody (Sigma #F1804, 1:1000), calnexin antibody (Santa Cruz #SC-6465, 1:500), Hoechst 33342 (ThermoFisher #H3570, 1:20000), donkey anti-mouse antibody, Alexa Fluor® 488 conjugate (ThermoFisher #A21202, 1:5000), and donkey anti-goat antibody, Alexa Fluor® 594 conjugate (ThermoFisher #A11058, 1:5000).

### Luciferase Assay

Luciferase assays were performed by using the Promega dual luciferase reporter assay kit as per the manufacturer’s instructions. Cells were transfected with Myrf-expressing plasmids, Myrf luciferase reporters, and pRL-TK (an internal control). Myrf luciferase reporters were generated by cloning Myrf ChIP-seq peaks^2^ into pGL3-promoter. The genomic coordinates of the luciferase reporters are as follows: Rffl (rn4 chr10:71034166-71034749), Tpra1 (rn4 chr4:123025896-123026295), 21 (rn4 chr1:23885816-23886215), and Sox10 (mm9 chr15:79023342-79024150).

### DNA Pulldown Assay

HEK293FT cells were transfected with Flag-Myrf or its variants. Upon cell lysis, cell lysate was cleared by centrifugation at 15,000 *g* for 20 min at 4 °C. Biotinylated duplex oligonucleotides were conjugated to Dynabeads (Invitrogen) in buffer A (5 mM Tris pH 8.0, 0.5 mM EDTA, 1 M NaCl). Oligonucleotide-conjugated beads were washed twice with 500 µl of buffer A and three times with buffer C (20 mM Tris pH 8.0, 1 mM EDTA, 10% glycerol, 1 mM DTT, 50 mM NaCl). 300 µg of cell lysate were incubated with oligonucleotide-conjugated beads in buffer C and sheared salmon sperm DNA (final concentration 0.2 mg/ml) for 20 min at room temperature with rotation. The mixture was spun down to separate the bead and sup fractions. The bead fraction was washed five times with 500 µl buffer C, and both fractions were analysed by immunoblotting. The DNA sequences used are as follows. The Myrf motif^15^ is underlined, and the mutated portions are indicated in bold.

WT:TGACTACCCCACAAGCTGGCACTGCCTGGCGCGGCCA

MU:TGACTACCCCACAAG**TCAACAT**TGCCTGGCGCGGCCA

## Electronic supplementary material

Supplementary information

## Data Availability

The datasets generated during and/or analysed during the current study are available from the corresponding author on reasonable request.
