# Supplementary material for: Elucidating the transactivation domain of the pleiotropic transcription factor Myrf
Source: Sci Rep. 2018 Aug 30;8:13075. doi: 10.1038/s41598-018-31477-4 (PMC6117317; doi:10.1038/s41598-018-31477-4)

Supplementary information

Elucidating the transactivation domain of the pleiotropic transcription factor Myrf

Jin-ok Choi[1,#], Chuandong Fan[1,#], Dongkyeong Kim[1], Mohamed Sharif[1], Hongjoo An[1], and Yungki Park[1,*]

[1]Hunter James Kelly Research Institute, Department of Biochemistry, Jacobs School of Medicine and Biomedical Sciences, SUNY Buffalo, Buffalo, NY 14203, USA
[#]Equal contribution
*To whom correspondence should be addressed.
Tel: 1-716-881-7579; Fax: 1-716-849-6651; Email: yungkipa@buffalo.edu

Supplemental Figure 1A

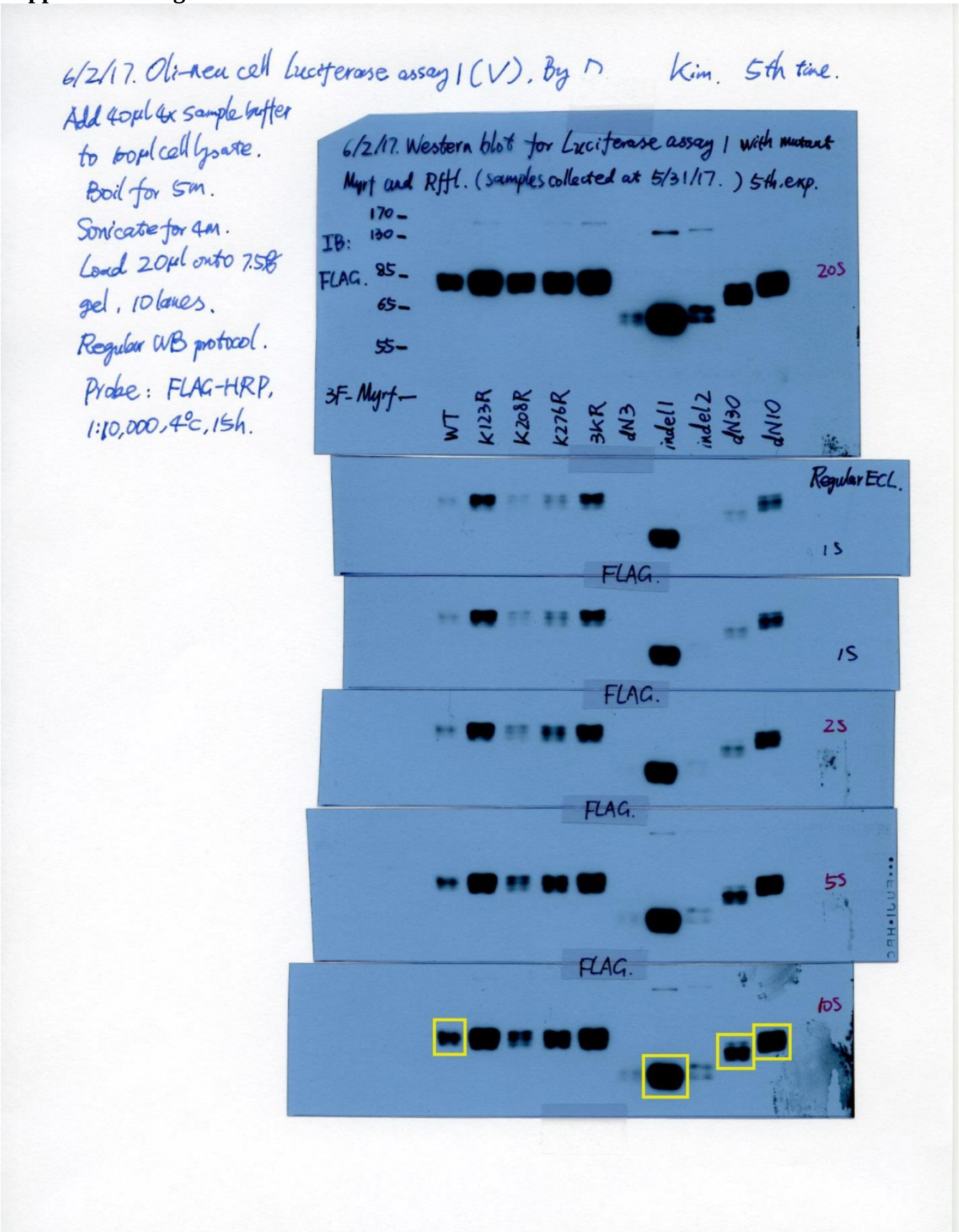

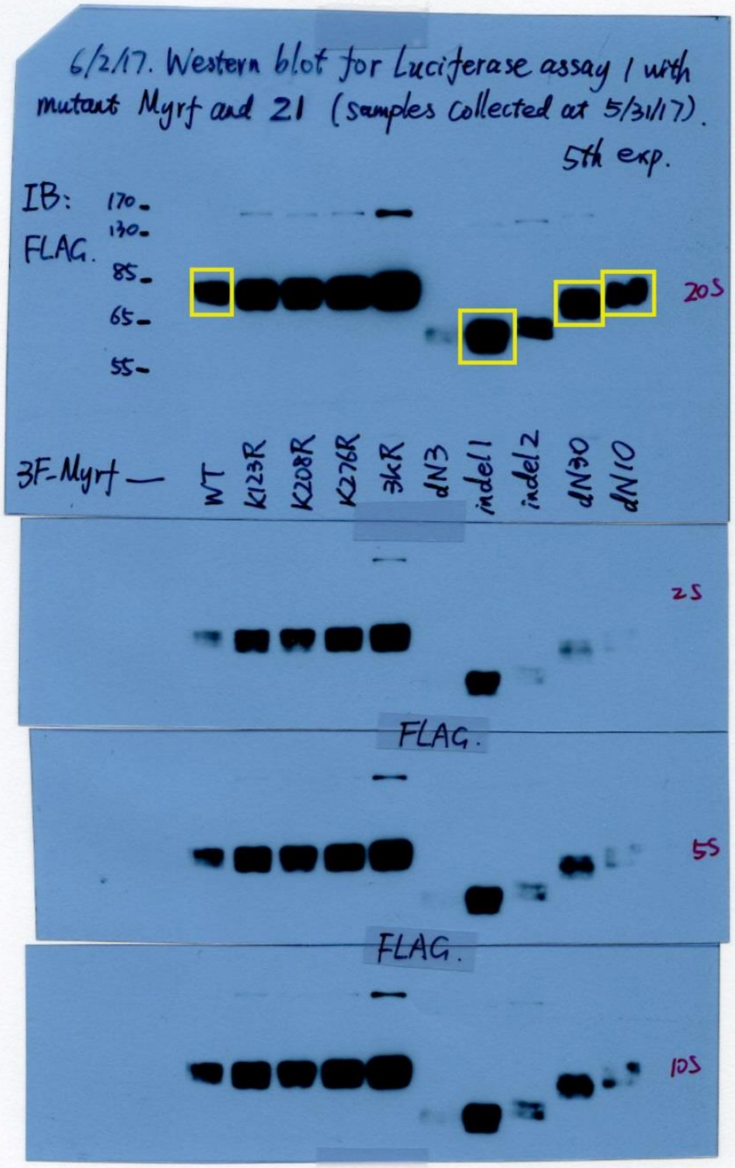
6/2/17. Western blot for Luciferase assay 1 with mutant Myrf and 21 (samples collected at 5/31/17).
5th exp.
IB: FLAG.
170
130
85
65
55
20S
3F-Myrf —
WT
K123R
K208R
K276R
3kR
dN3
indel1
indel2
dN30
dN10
2S
FLAG.
5S
FLAG.
10S

6/2/17. Western blot for Luciferase assay 1 with mutant Myrf and Rfl(, 5th exp. Reprobe α-tubulin and 21.

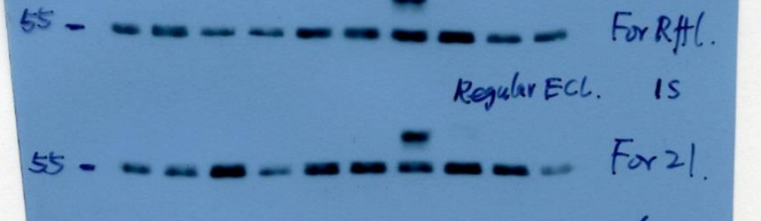

Anti α-tubulin, 1:10,000, RT, 30M. GaM 1:5000, RT, 60m. in 3% BSA.

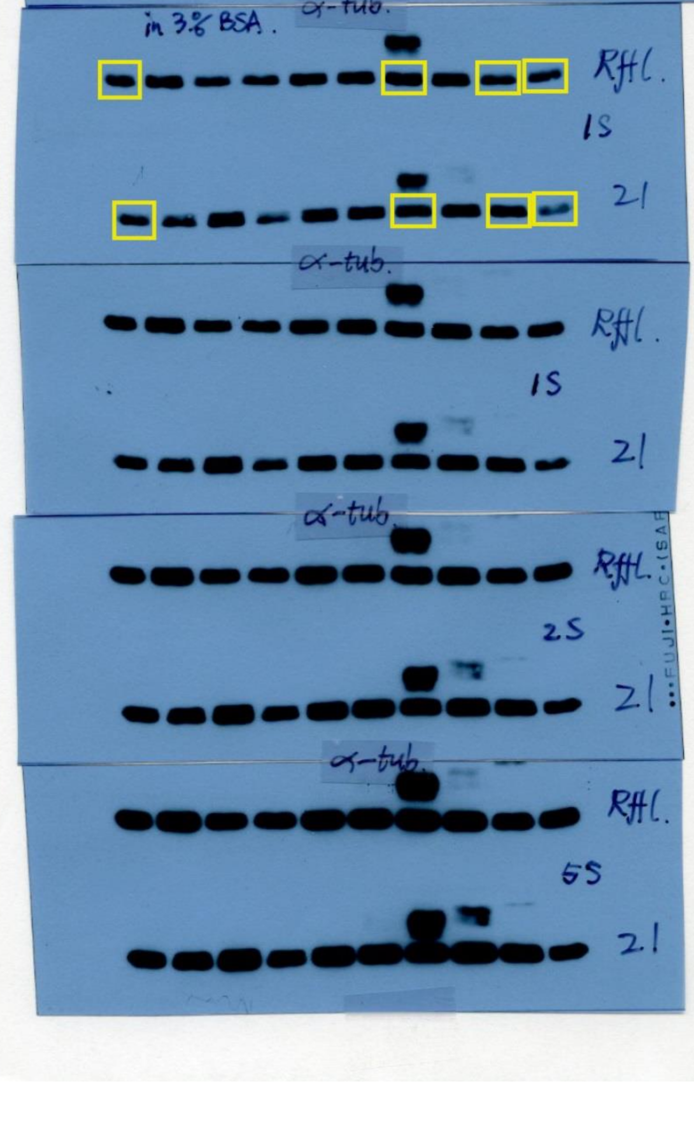

Supplemental Figure 1B

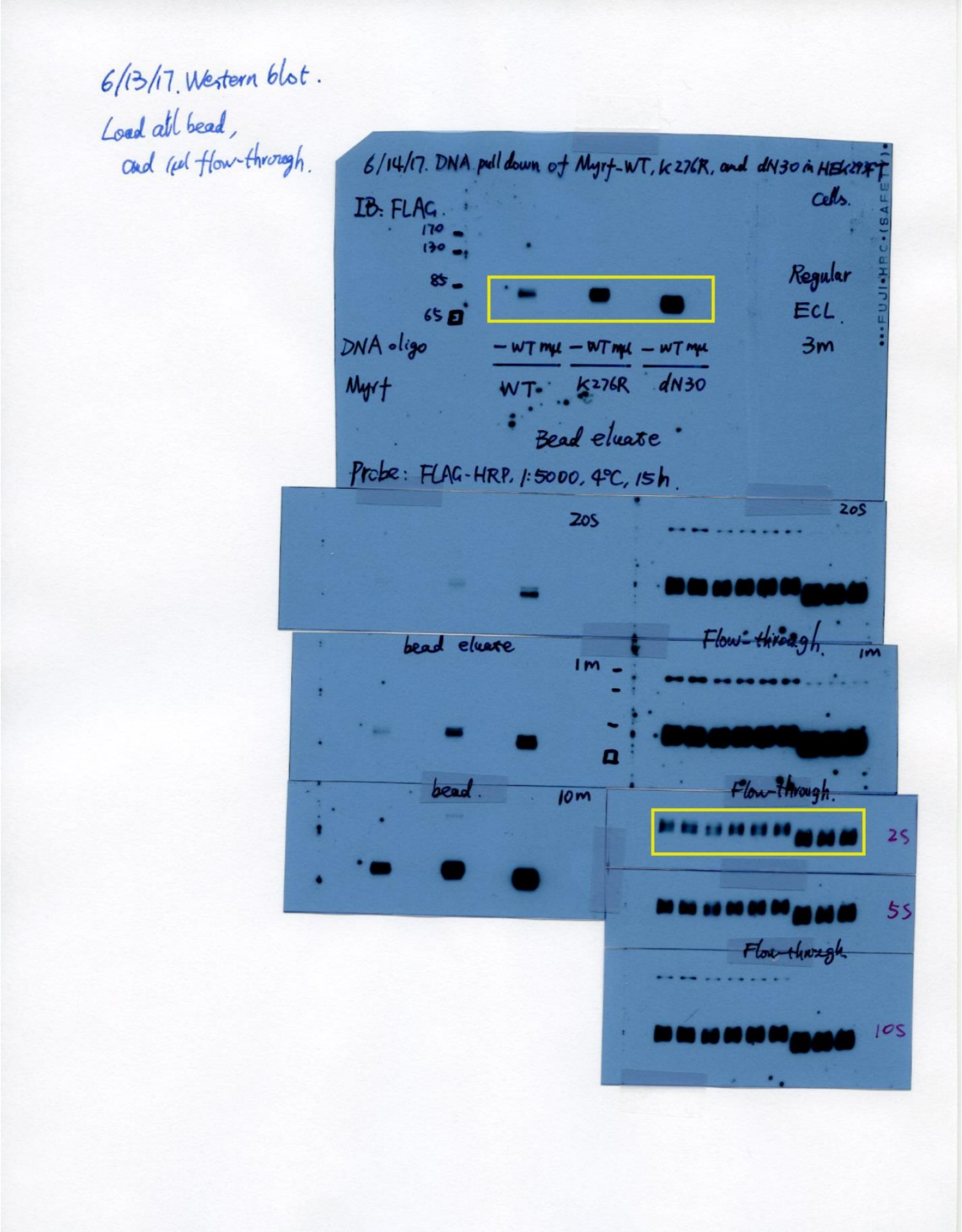

Supplemental Figure 2

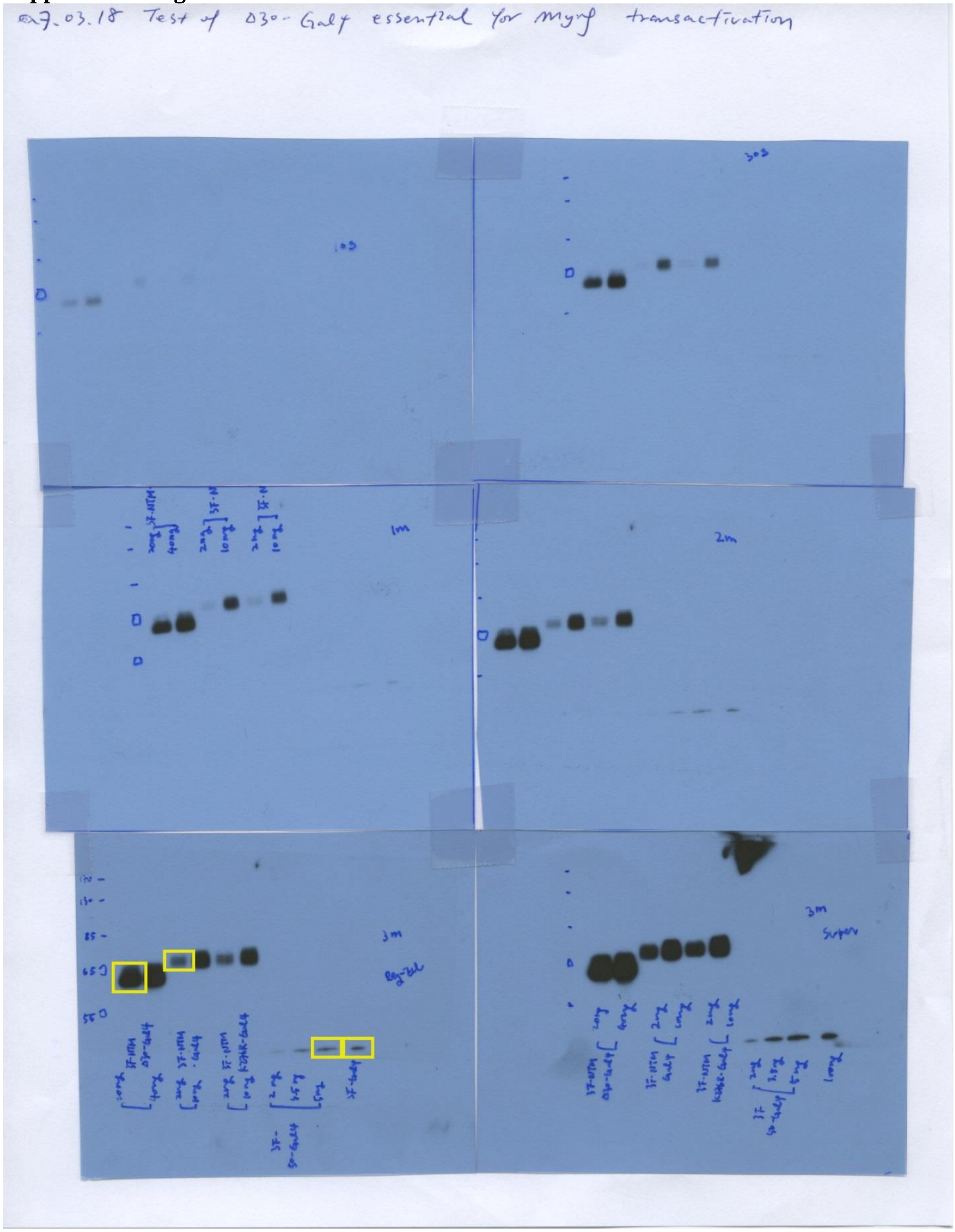

07.06.18 Test of Δ30-Gal4 essential for Myrf transactivation

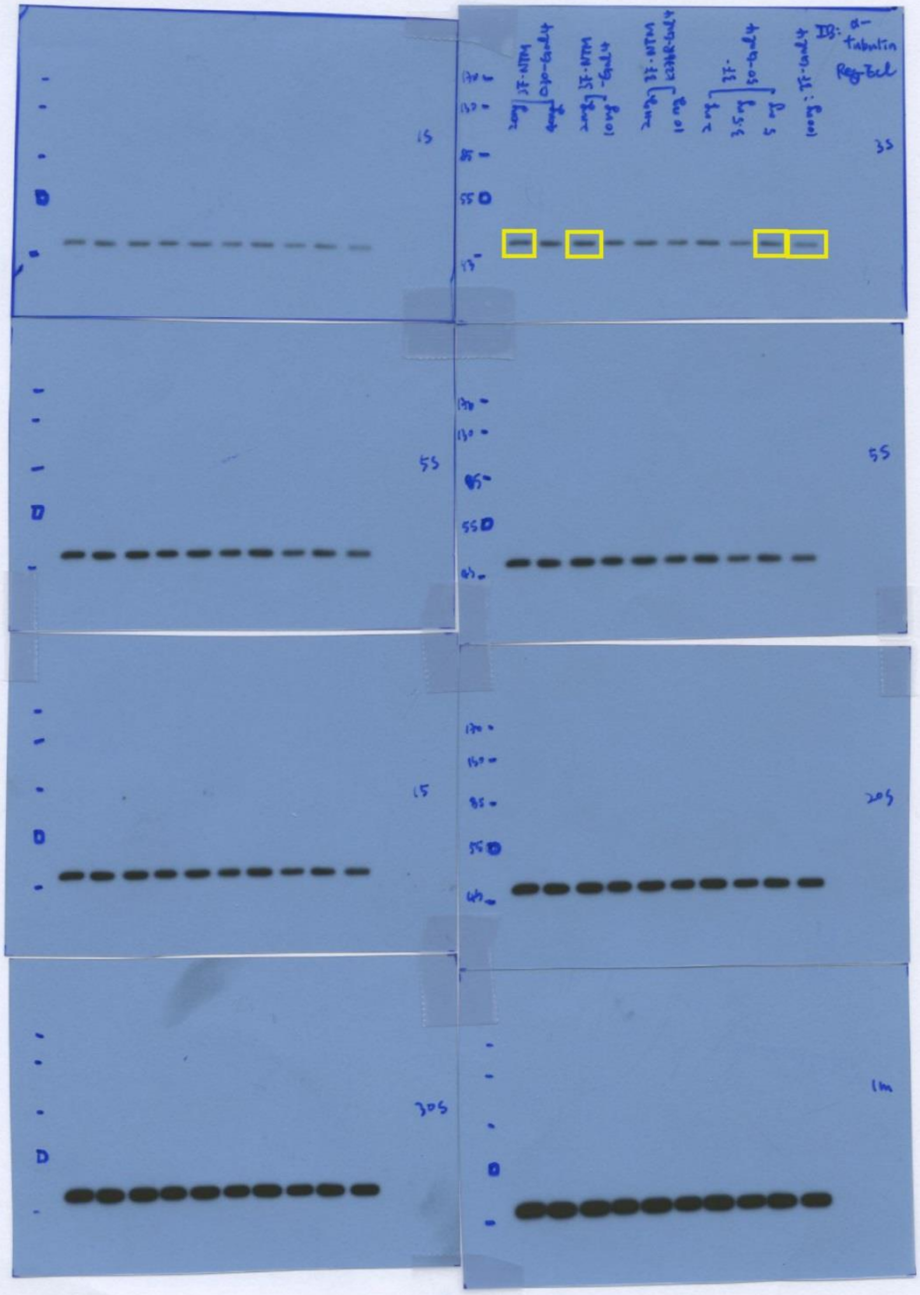

2018. 07. 12 NTM 30 domain of Myrf transcription on HEK293FT

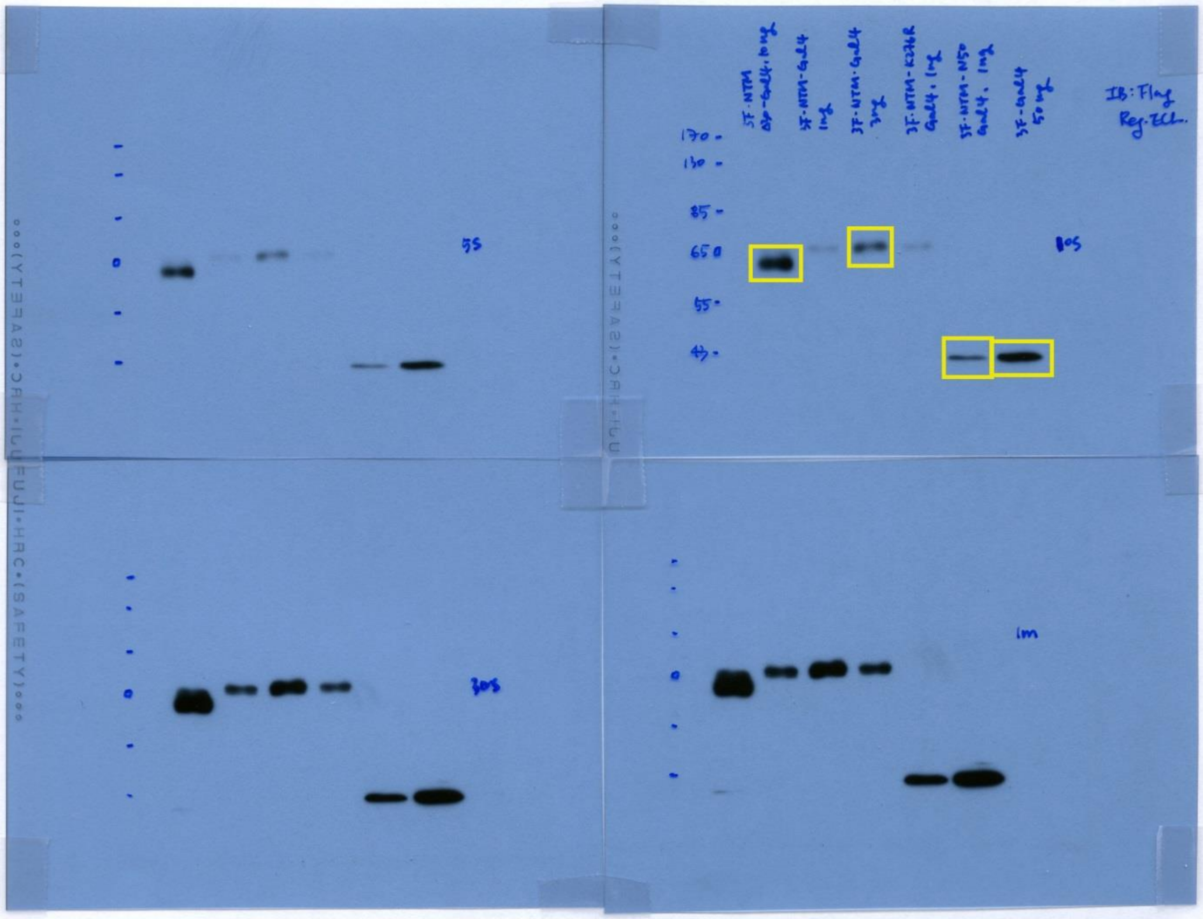

180713 Test NTM 30 domain for Myrf transactivation on HEK293FT - α-Tubulin

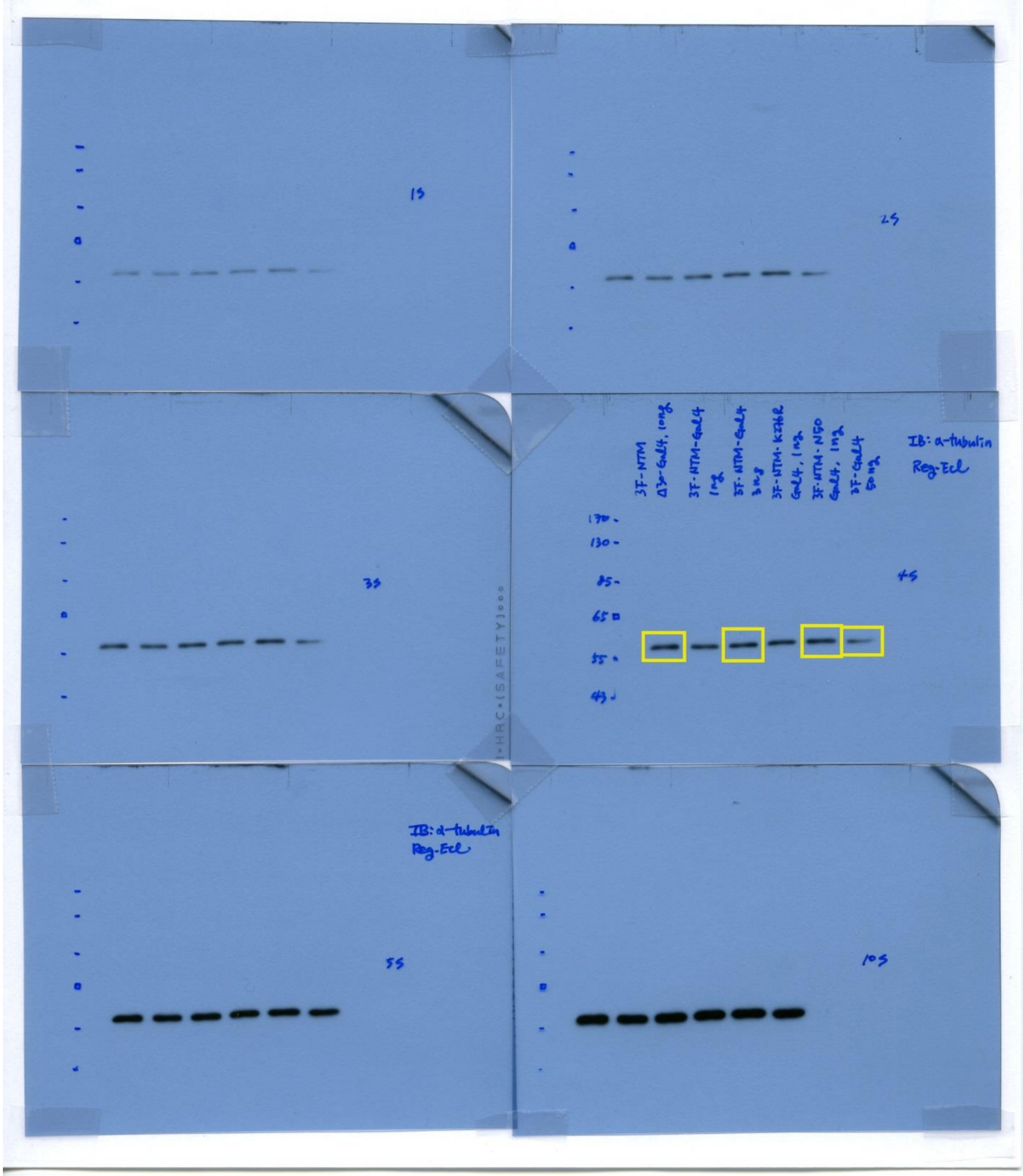

Supplemental Figure 3A

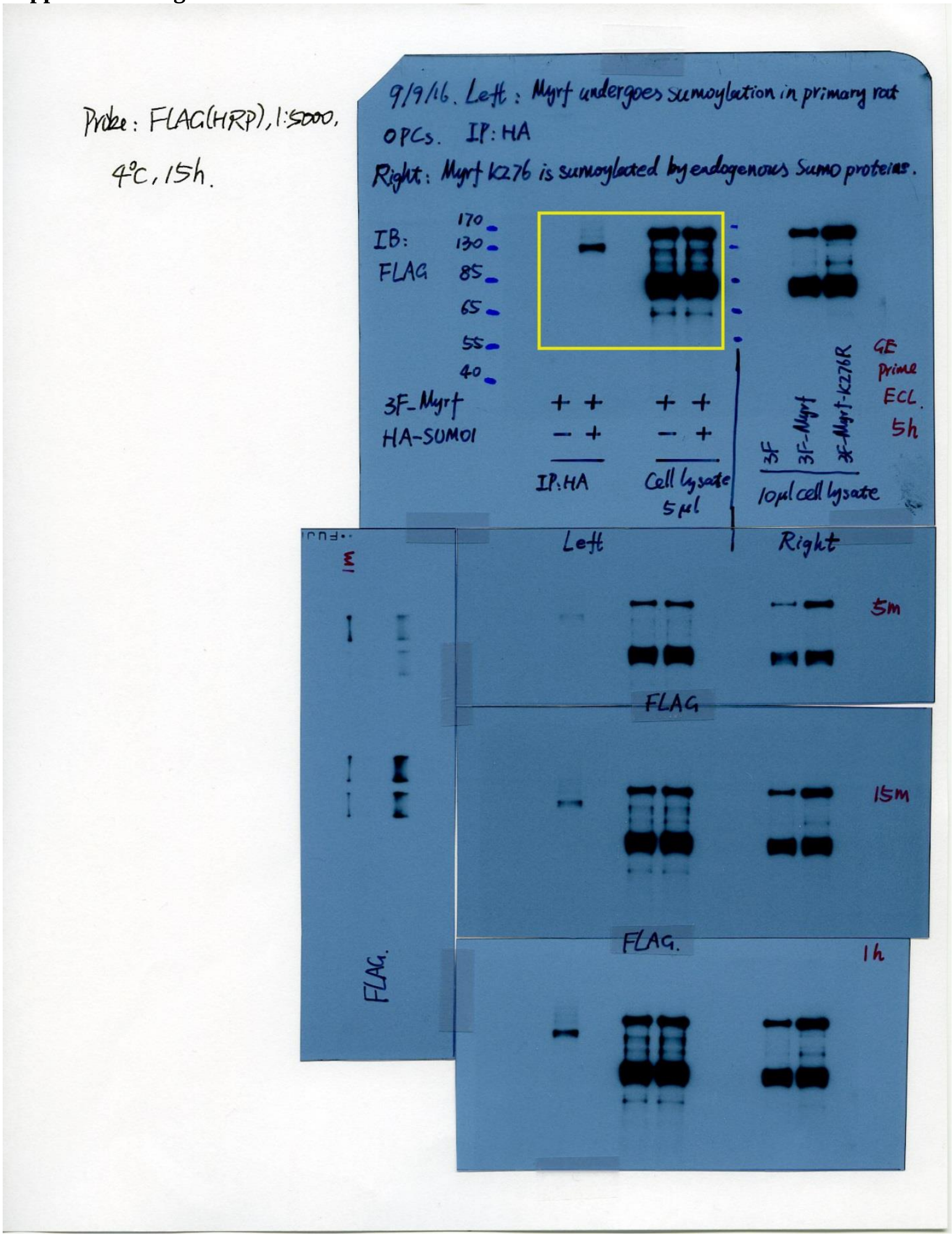

Supplemental Figure 3B

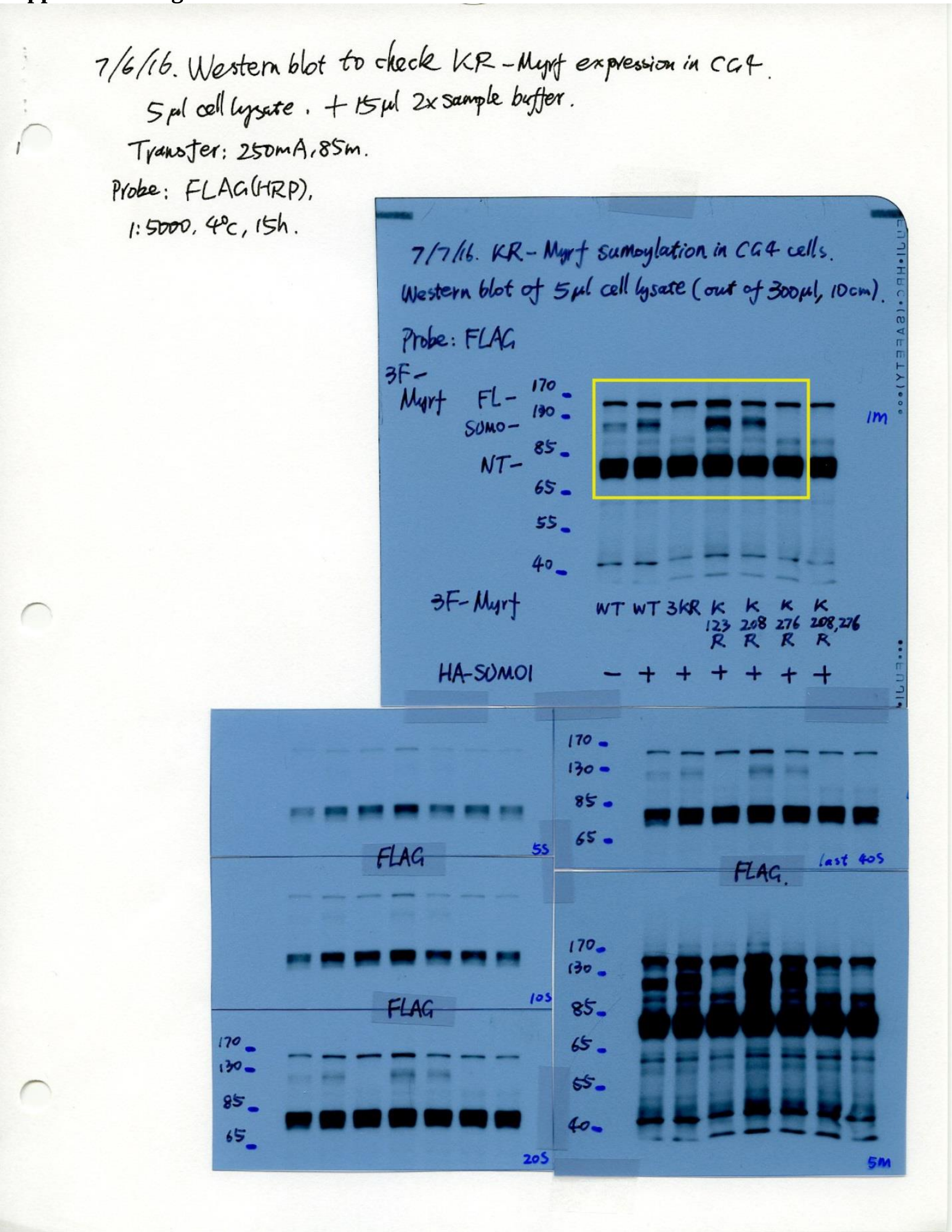

7/7/16. Western blot for IP: HA.

Add 40µl 2x sample buffer to beads, load 20µl to 7.5% gel.

Let 65kDa size marker hit bottom.

Transfer: 250mA, 85m.

Probe: FLAG (HRP), 1:5000, 4°C, 15h.

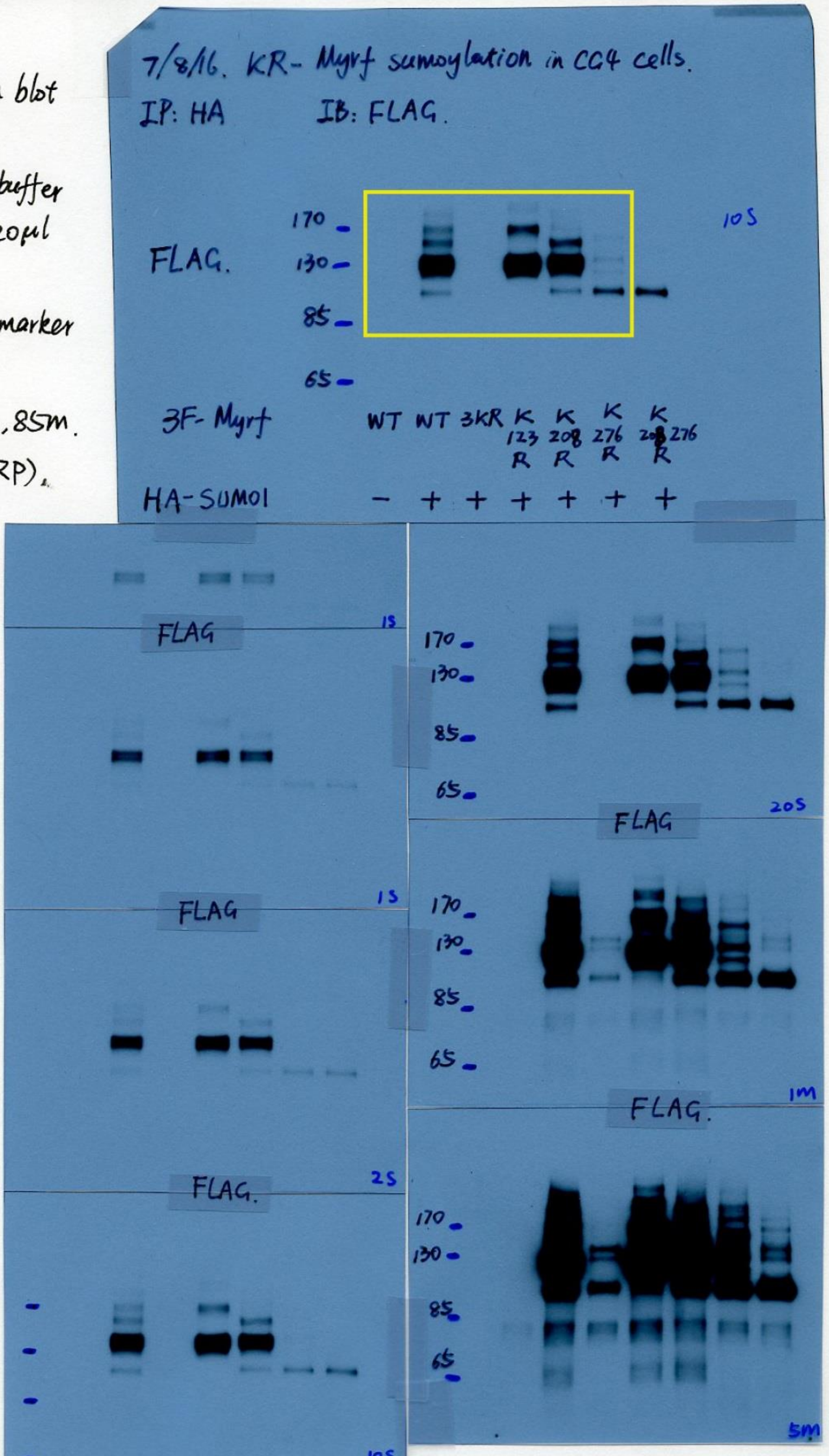

Supplemental Figure 3C

6/30/16. Western blot of the cell lysate, to check KR-Myrf expression in HEK cells.

2μl cell lysate + 18μl 2x sample buffer. 7.5% gel, 60, 120, 200V.

Transfer: 250mA, 65m.

Probe: FLAG(HRP), 1:5000, 4°C, 3.5h. Regular ECL.

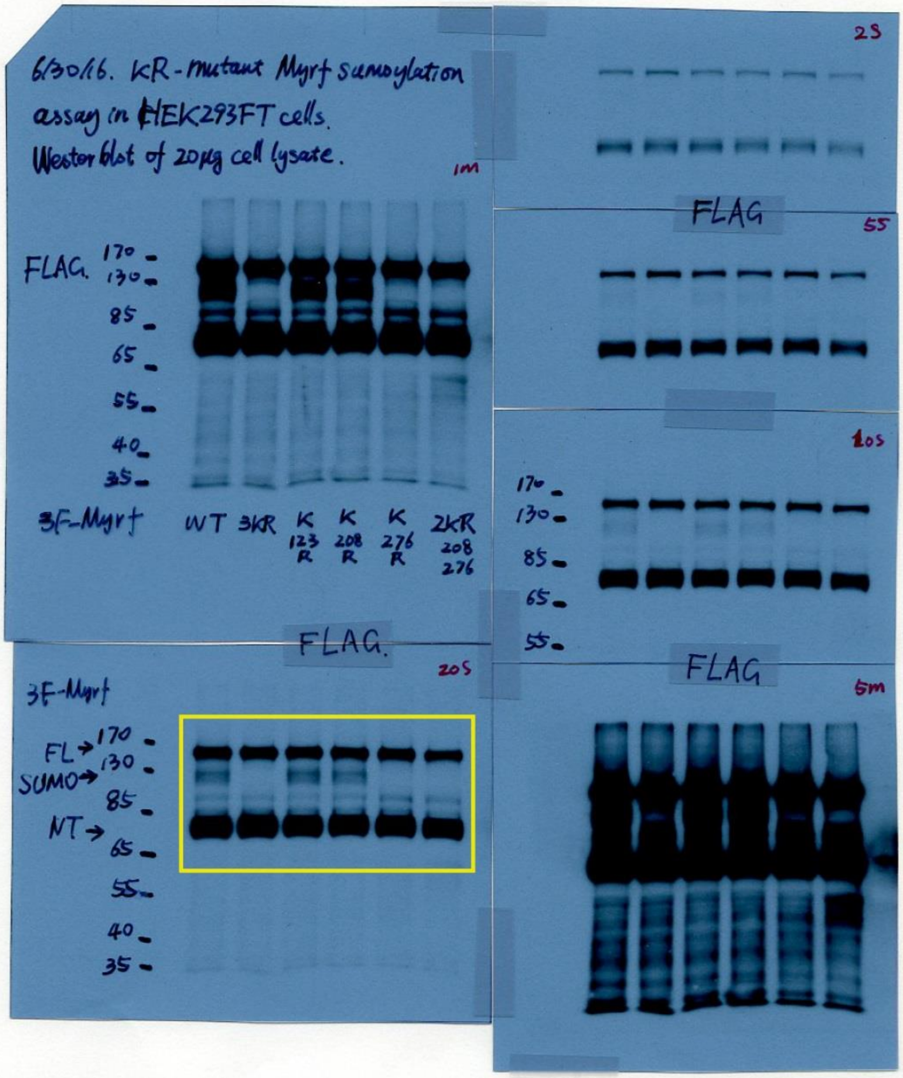

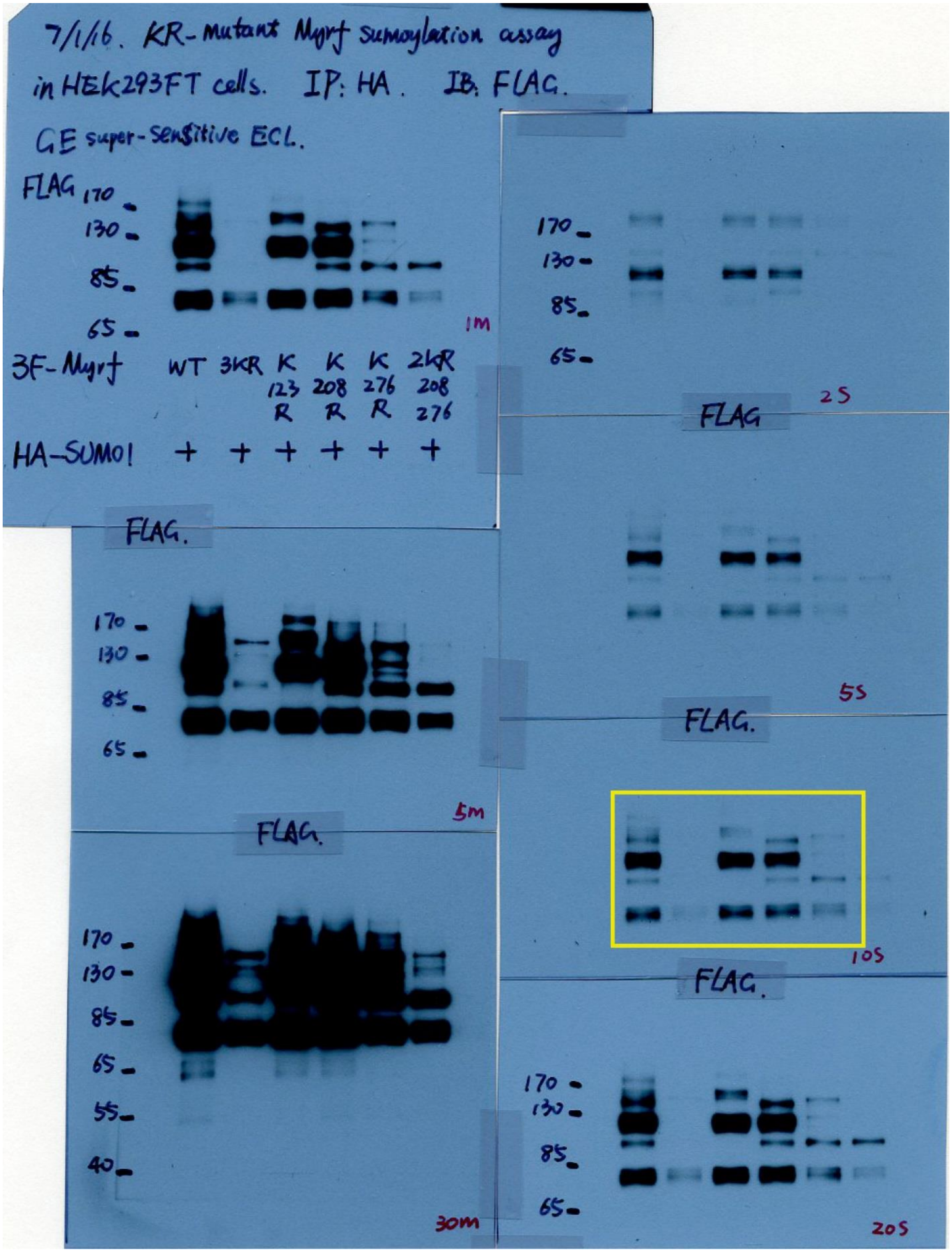
7/1/16. KR-mutant Myrf sumoylation assay
in HEK293FT cells. IP: HA. IB: FLAG.
GE super-sensitive ECL.
FLAG
170
130
85
65
1M
3F-Myrf WT 3KR K123R K208R K276R 2KR 208 276
HA-SUMO1 + + + + + +
FLAG.
170
130
85
65
5m
FLAG.
170
130
85
65
55
40
30m
170
130
85
65
2S
FLAG
5S
FLAG.
10S
FLAG.
170
130
85
65
20S

1/12/17 Reprobe HA, after SUMO1 probing.

Anti-HA, (HRP), cell signaling, 6E2, #2999, 1:10,000, 4°C, 15h.

1/13/17. KR-mutant 3F-Myrf sumoylation assay in HEK293FT cells. Reprobe HA after SUMO1 probing.

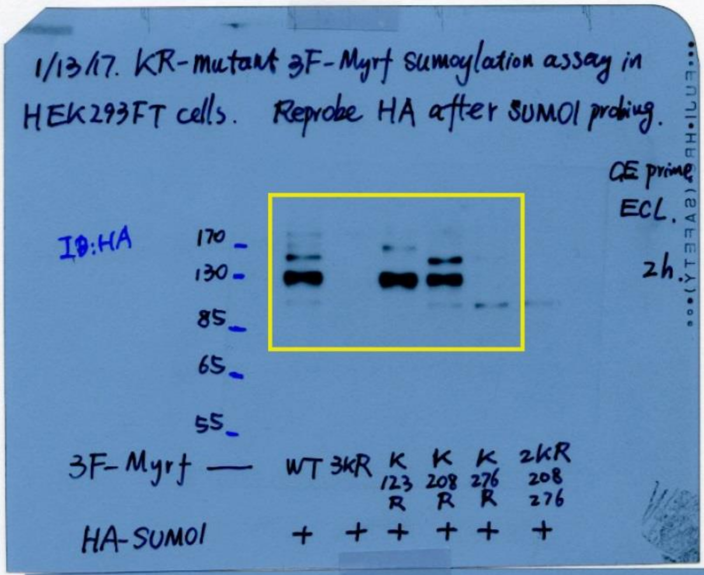

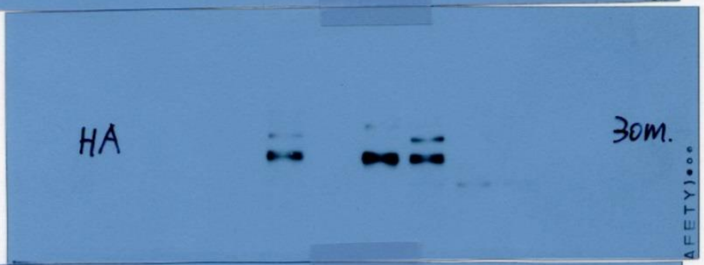

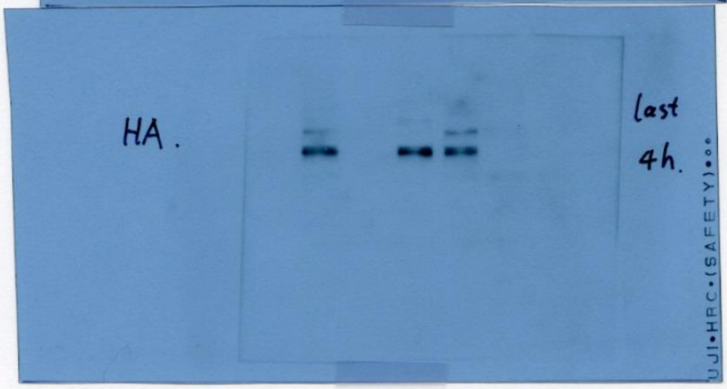

Supplemental Figure 3D

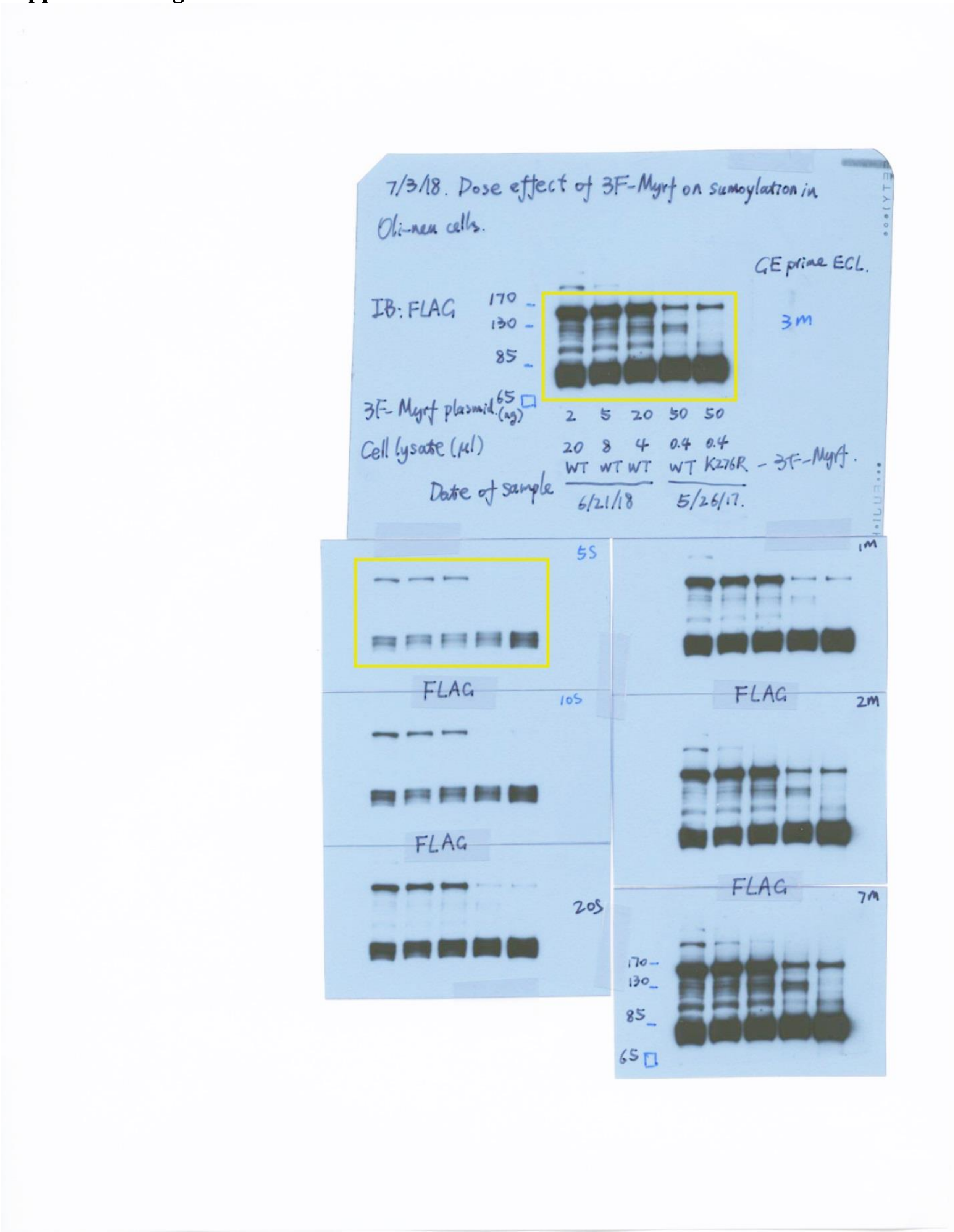

20180702 Diluete protein samples to see sumoylation

sample volume Out of 140 ul from 3.5 cm dish, 6/21/2018

	sample vol	Dilution times	2xsample buffer	Final volume	Name of Final sample
3	20	1	0	20	3d
2	8	2.5	12	20	2d
1	4	5	16	20	1d

sample volume Out of 170 ul from 3.5 cm dish, 5/26/201~~8~~7, Half-life of 3F-Myrf-wt and K276R Box 1, -80°C.

				loading		
1	4	50	196	20	o1d	3F-Myrf-wt
6	4	50	196	20	o6d	3F-Myrf-K276R

WB: 7.5% gel, 10 lanes.

IB: (1) FLAG-HRP, 1:5000, in 5% milk, 4°C, 15h.

(2) α-tubulin, 1:10,000, in 5% milk, with GαM-HRP, 1:5000, RT, 1h.

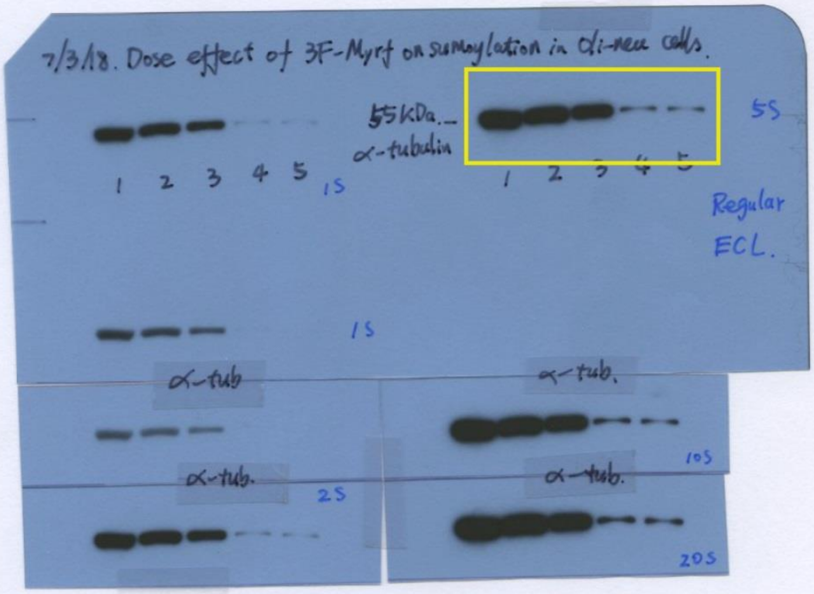

Supplemental Figure 4A

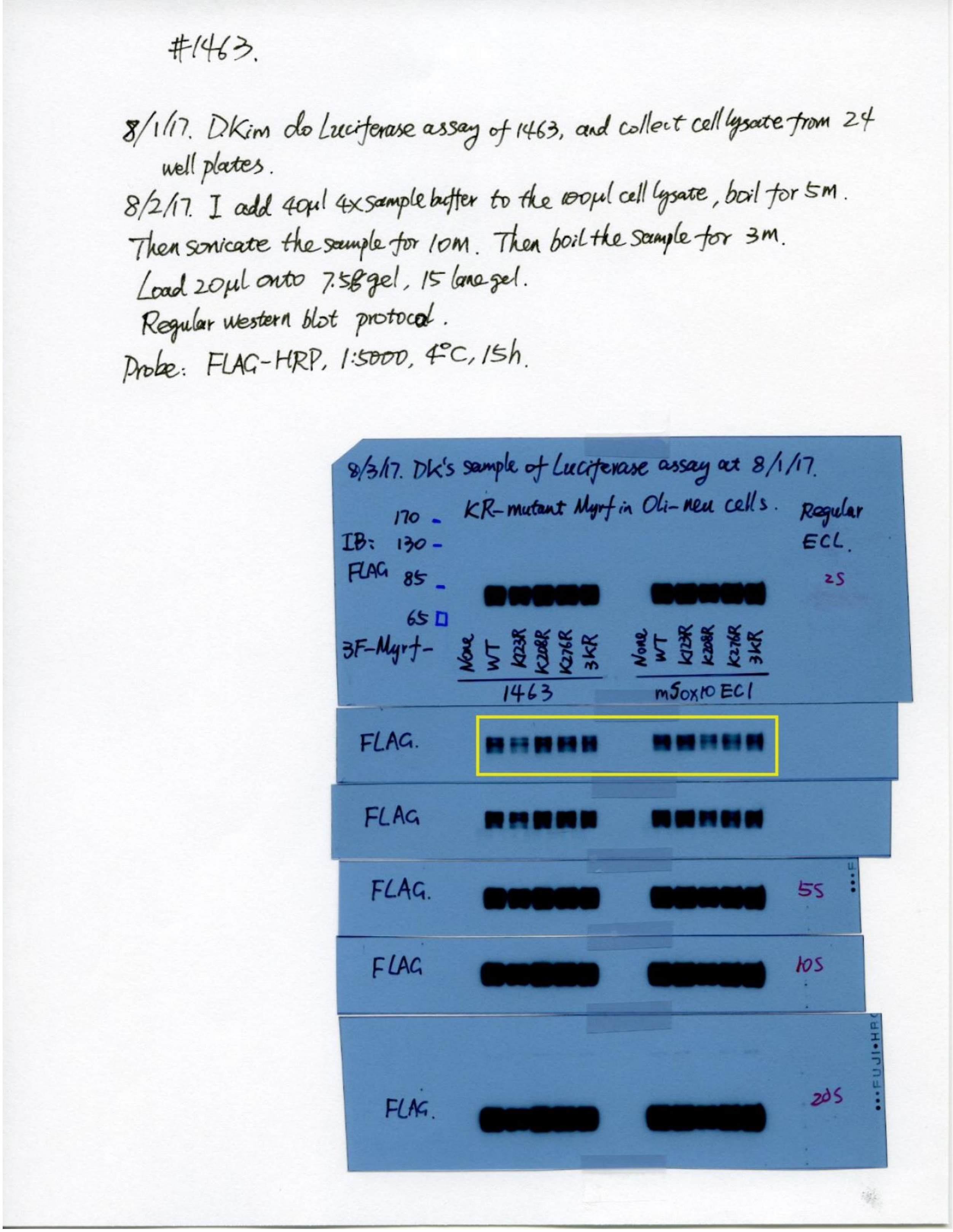

8/3/17. Reprobe α-tubulin, 1:5000, with GaM-HRP, 1:2500,
RT, 5m.

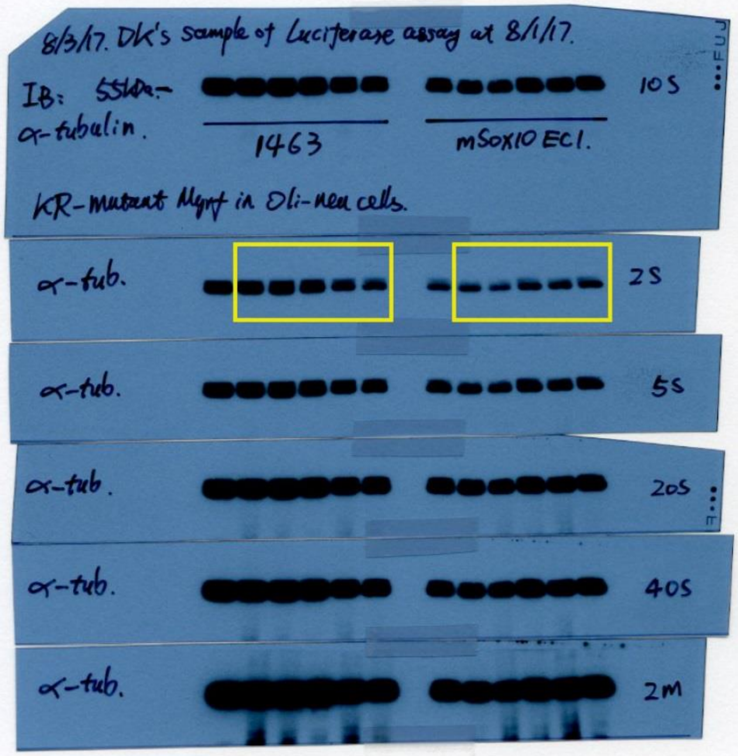

Supplemental Figure 4B

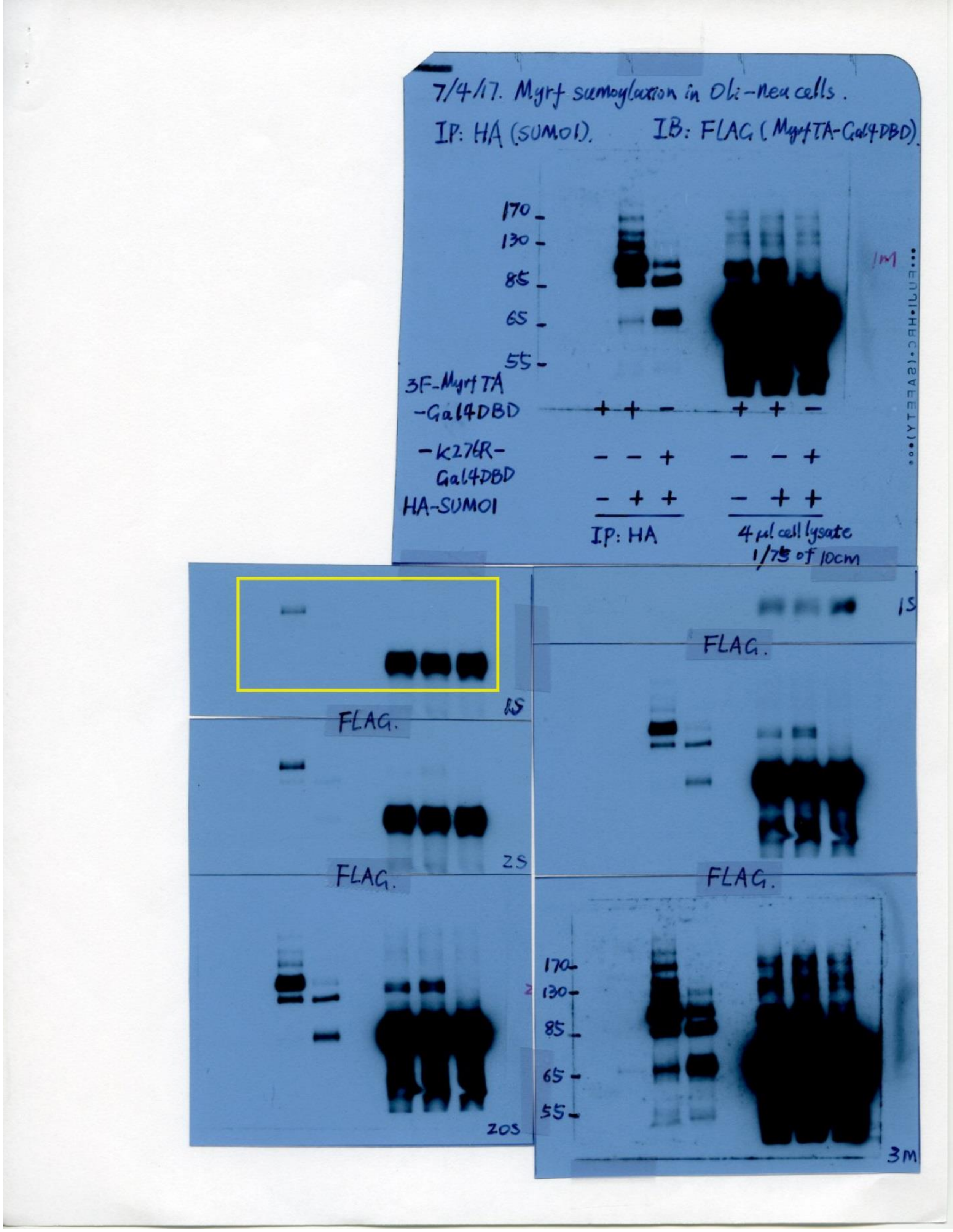

Supplemental Figure 4C

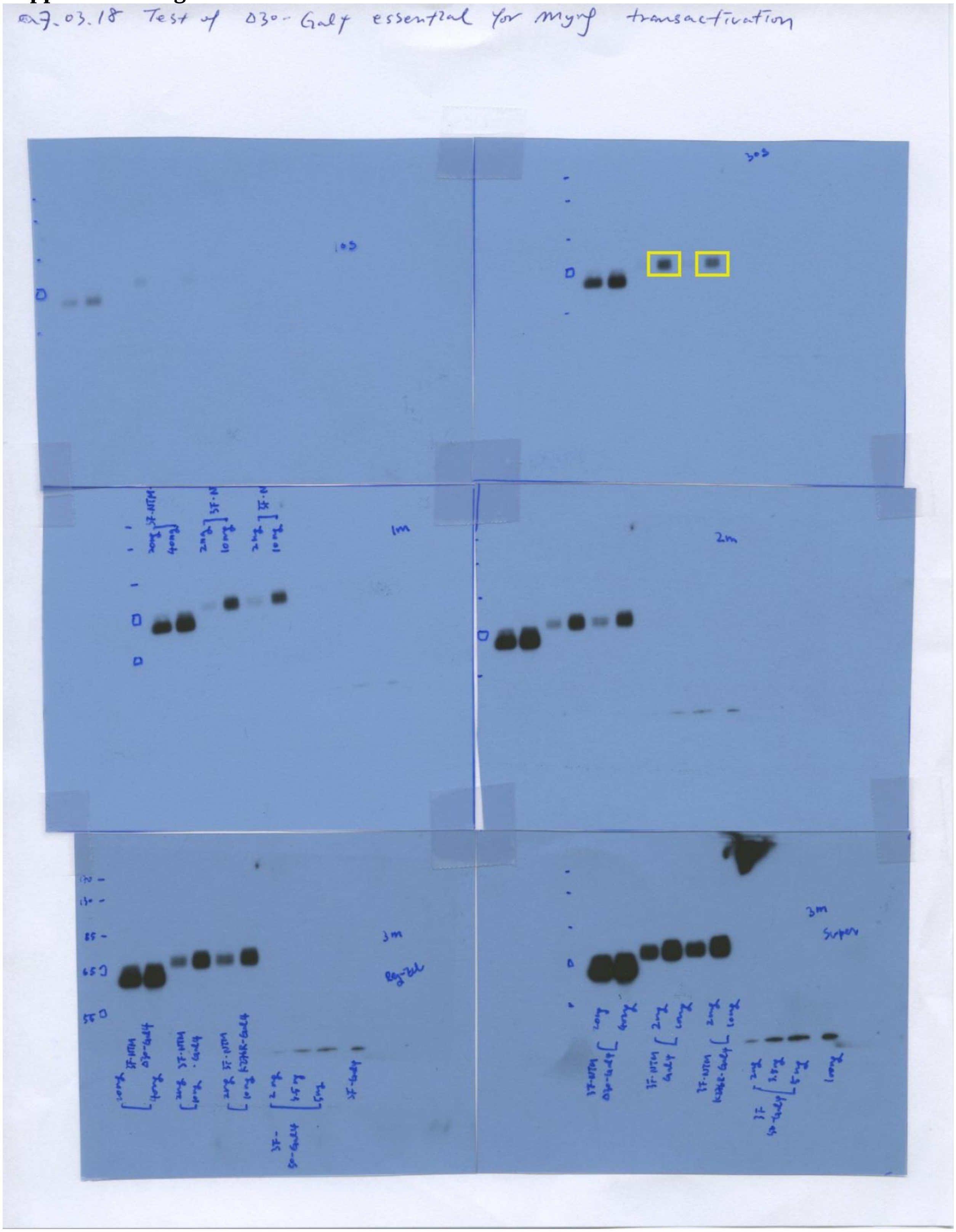

07.06.18 Test of Δ30-Gal4 essential for Myf transactivation

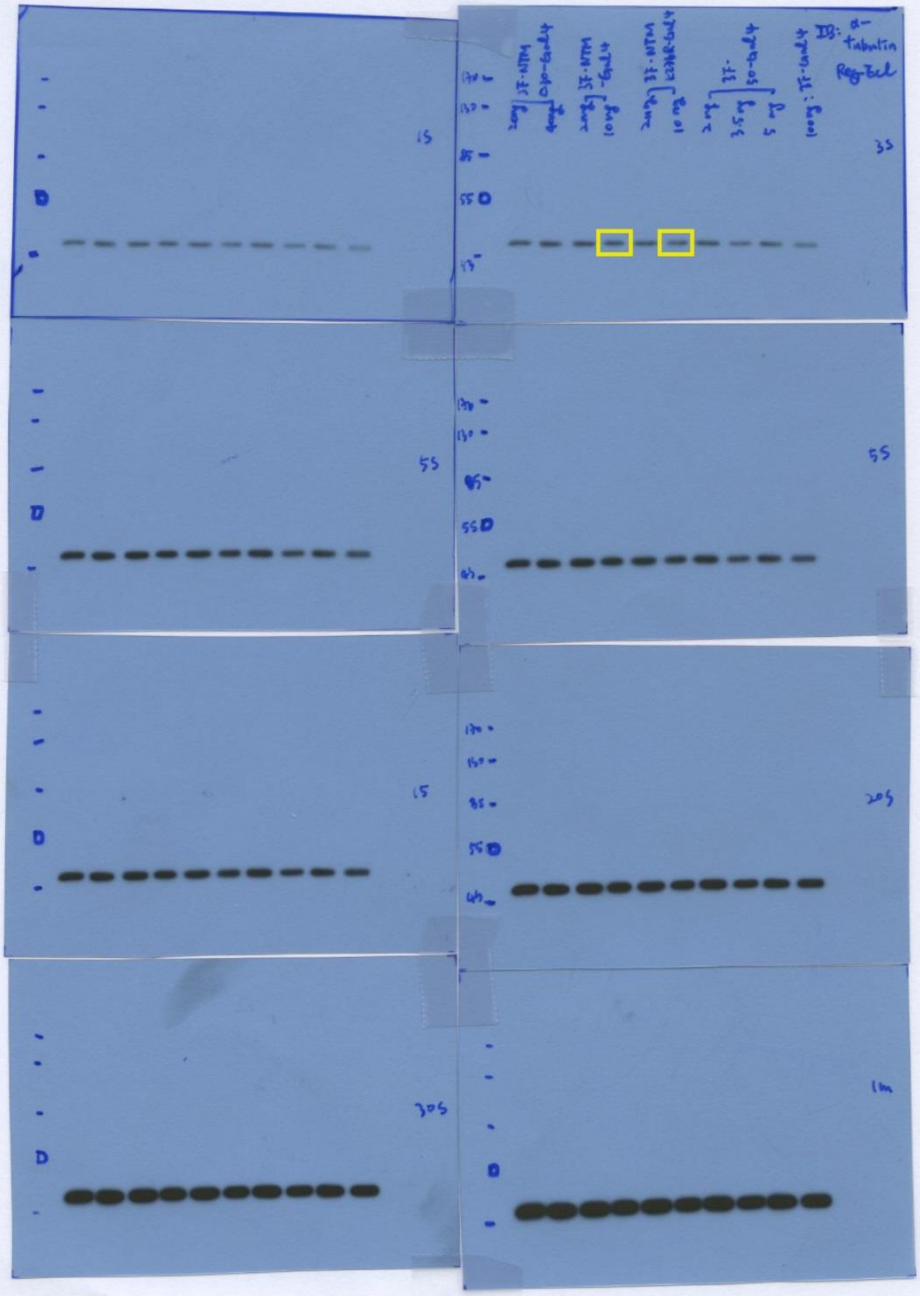

2018.07.12 NTM 30 domain of Myrf transcription on HEK293FT

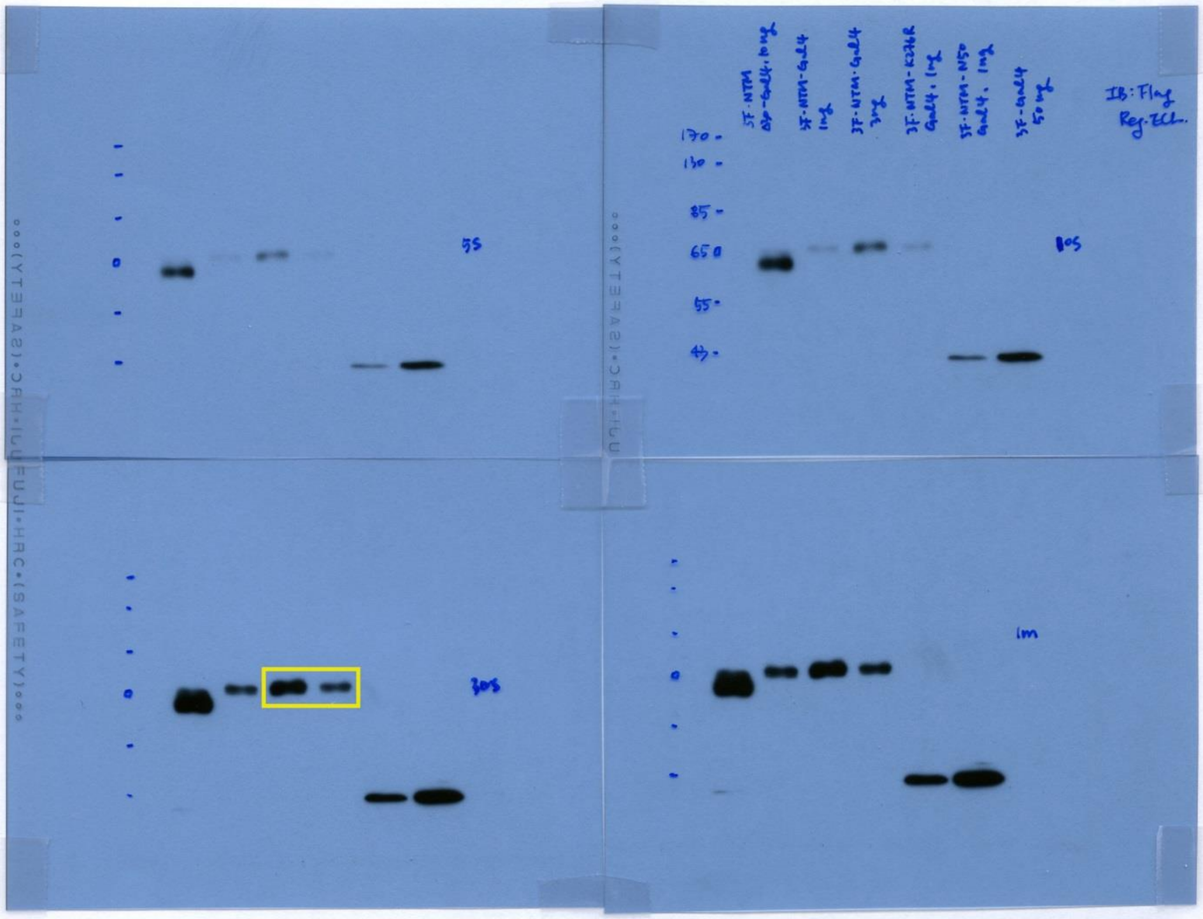

180713 Test NTM 30 domain for Myrf transactivation on HEK293FT - α-Tubulin

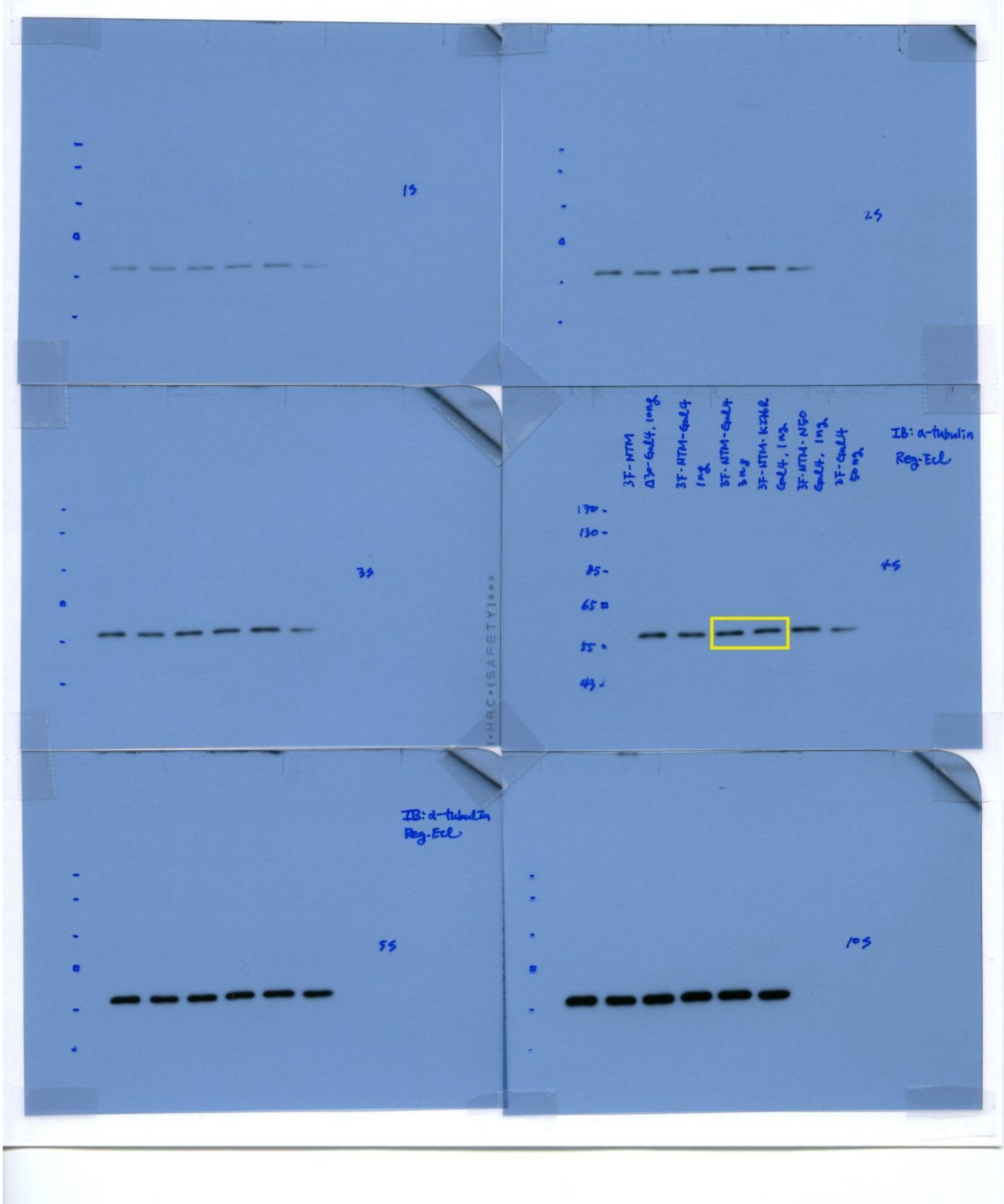

Supplemental Figure 4D

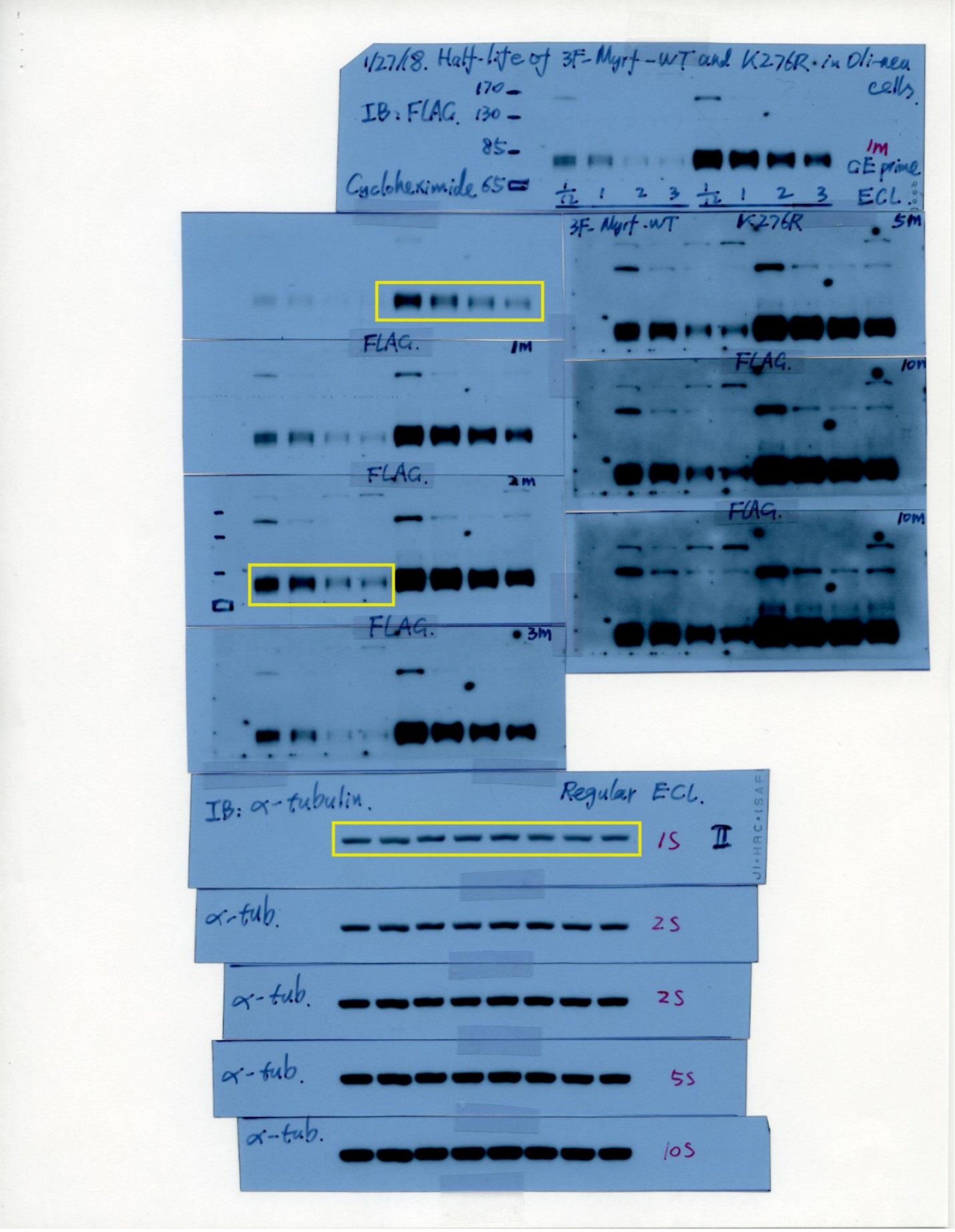

Supplement: Supplementary file 1 — Supplementary information [file 41598_2018_31477_MOESM1_ESM.pdf]
